# Investigating two mobile just-in-time adaptive interventions to foster psychological resilience: research protocol of the DynaM-INT study

**DOI:** 10.1186/s40359-023-01249-5

**Published:** 2023-08-25

**Authors:** S. A. Bögemann, A. Riepenhausen, L. M. C. Puhlmann, S. Bar, E. J. C. Hermsen, J. Mituniewicz, Z. C. Reppmann, A. Uściƚko, J. M. C. van Leeuwen, C. Wackerhagen, K. S. L. Yuen, M. Zerban, J. Weermeijer, M. A. Marciniak, N. Mor, A. van Kraaij, G. Köber, S. Pooseh, P. Koval, A. Arias-Vásquez, H. Binder, W. De Raedt, B. Kleim, I. Myin-Germeys, K. Roelofs, J. Timmer, O. Tüscher, T. Hendler, D. Kobylińska, I. M. Veer, R. Kalisch, E. J. Hermans, H. Walter

**Affiliations:** 1 Donders Institute for Brain, Cognition and Behaviour, Radboud University Medical Center, Kapittelweg 29, Nijmegen, 6525 EN The Netherlands; 2 Research Division of Mind and Brain, Department of Psychiatry and Neurosciences CCM, Charité - Universitätsmedizin Berlin, Corporate Member of Freie Universität Berlin, Humboldt-Universität Zu Berlin, and Berlin Institute of Health, Berlin, Germany; 3 Faculty of Philosophy, Berlin School of Mind and Brain, Humboldt-Universität Zu Berlin, Berlin, Germany; 4 Leibniz Institute for Resilience Research (LIR), Mainz, Germany; 5 Max Planck Institute for Human Cognitive and Brain Sciences, Leipzig, Germany; 6 Sagol Brain Institute, Tel Aviv Sourasky Medical Center, Tel Aviv, Israel; 7 Faculty of Psychology, University of Warsaw, Warsaw, Poland; 8 Neuroimaging Center (NIC), Focus Program Translational Neuroscience (FTN), Johannes Gutenberg University Medical Center, Mainz, Germany; 9 Center for Contextual Psychiatry, Department of Neurosciences, KU Leuven, Louvain, Belgium; 10 Division of Experimental Psychopathology and Psychotherapy, Department of Psychology, University of Zurich, Zurich, Switzerland; 11 Department of Psychiatry, Psychotherapy and Psychosomatics, Psychiatric University Hospital (PUK), University of Zurich, Zurich, Switzerland; 12 Sackler Faculty of Medicine, Tel Aviv University, Tel Aviv, Israel; 13 OnePlanet Research Center, Wageningen, The Netherlands; 14 Institute of Medical Biometry and Statistics, Faculty of Medicine and Medical Center, University of Freiburg, Freiburg, Germany; 15 Freiburg Center for Data Analysis and Modelling, University of Freiburg, Freiburg, Germany; 16 Melbourne School of Psychological Sciences, The University of Melbourne, Vic, 3010 Australia; 17 Life Sciences Department, Imec, Louvain, Belgium; 18 Center for Cognitive Neuroimaging, Donders Institute for Brain Cognition and Behaviour, Radboud University, Nijmegen, The Netherlands; 19 Behavioural Science Institute, Radboud University, Nijmegen, The Netherlands; 20 Institute of Physics, University of Freiburg, Freiburg, Germany; 21 Signalling Research Centres BIOSS and CIBSS, University of Freiburg, Freiburg, Germany; 22 Department of Psychiatry and Psychotherapy, Johannes Gutenberg University Medical Center, Mainz, Germany; 23 School of Psychological Science, Tel Aviv University, Tel Aviv, Israel; 24 Sagol School of Neuroscience, Tel Aviv University, Tel Aviv, Israel; 25 Department of Developmental Psychology, University of Amsterdam, Amsterdam, The Netherlands

**Keywords:** Resilience, Stress, Resilience factors, Mental health, Longitudinal, Prospective, Ecological momentary assessment, Ecological momentary intervention, Reappraisal, Mental imagery

## Abstract

**Background:**

Stress-related disorders such as anxiety and depression are highly prevalent and cause a tremendous burden for affected individuals and society. In order to improve prevention strategies, knowledge regarding resilience mechanisms and ways to boost them is highly needed. In the Dynamic Modelling of Resilience – interventional multicenter study (DynaM-INT), we will conduct a large-scale feasibility and preliminary efficacy test for two mobile- and wearable-based just-in-time adaptive interventions (JITAIs), designed to target putative resilience mechanisms. Deep participant phenotyping at baseline serves to identify individual predictors for intervention success in terms of target engagement and stress resilience.

**Methods:**

DynaM-INT aims to recruit *N* = 250 healthy but vulnerable young adults in the transition phase between adolescence and adulthood (18–27 years) across five research sites (Berlin, Mainz, Nijmegen, Tel Aviv, and Warsaw). Participants are included if they report at least three negative burdensome past life events and show increased levels of internalizing symptoms while not being affected by any major mental disorder. Participants are characterized in a multimodal baseline phase, which includes neuropsychological tests, neuroimaging, bio-samples, sociodemographic and psychological questionnaires, a video-recorded interview, as well as ecological momentary assessments (EMA) and ecological physiological assessments (EPA).

Subsequently, participants are randomly assigned to one of two ecological momentary interventions (EMIs), targeting either positive cognitive reappraisal or reward sensitivity. During the following intervention phase, participants' stress responses are tracked using EMA and EPA, and JITAIs are triggered if an individually calibrated stress threshold is crossed. In a three-month-long follow-up phase, parts of the baseline characterization phase are repeated. Throughout the entire study, stressor exposure and mental health are regularly monitored to calculate stressor reactivity as a proxy for outcome resilience. The online monitoring questionnaires and the repetition of the baseline questionnaires also serve to assess target engagement.

**Discussion:**

The DynaM-INT study intends to advance the field of resilience research by feasibility-testing two new mechanistically targeted JITAIs that aim at increasing individual stress resilience and identifying predictors for successful intervention response. Determining these predictors is an important step toward future randomized controlled trials to establish the efficacy of these interventions.

**Supplementary Information:**

The online version contains supplementary material available at 10.1186/s40359-023-01249-5.

## Introduction

### Background

Stress-related mental disorders such as depression and anxiety disorders reside among the leading causes for disability worldwide [1–3] and cause a considerable burden to affected individuals, society, and the economy [4]. The general prevalence of mental disorders is particularly high in late teens and young adults in their twenties [5], with depression and anxiety showing a high rate of recurrence or persistence [6]. Although the link between stress and mental disorders has been well known for quite some time, the prevalence of stress-related disorders has not decreased during the last years [7]. Next to a failure to correctly implement clinical practice guidelines, one likely cause is the lack of appropriate and accessible prevention programs [7]. To inform prevention programs and help identifying possible prevention targets, research should ideally not only investigate contributing factors and mechanisms related to vulnerability, dysfunction, and psychopathology, but also investigate resilience, in order to identify factors and mechanisms that help people to stay healthy despite experiencing adversity [8].

Resilience can be defined as sustained or quickly recovering good mental health during and after experiencing adversity [9, 10]. This definition of resilience as an outcome rather than a trait reflects the difficulty to individually predict good long-term mental health responses to stressor exposure from a person’s stable features or predispositions and acknowledges that staying mentally healthy appears to result from putatively dynamic and complex processes allowing successful adaptation to stressors [8, 10–14]. These processes are not only determined by individual predisposing factors (so-called ‘resilience factors’, e.g., a certain genotype, stable personality traits, or beliefs) but also by characteristics specific to the adverse events or circumstances and an interplay between the two, and they involve the activation of protective mechanisms (‘resilience mechanisms’) at the level of the individual or the environment. Defining resilience as an outcome implies that resilience research should make use of longitudinal study designs, assessing adversity as well as mental health at several time points to capture the dynamic nature of occurring stressors and the possible subsequent changes in mental health [8, 10]. Another necessary element of resilience studies are assessments of resilience factors or mechanisms that can be linked to the outcome and which should ideally also be examined repeatedly, to thus uncover processes of adaptation [8].

Although some resilience factors are quite stable and will (mostly) not change much over the course of life (e.g., one’s genotype), other resilience factors are malleable and can undergo change, for example, triggered by the experience of adversity itself (e.g., one’s individual repertoire of emotion regulation strategies, which might increase after learning a new strategy during a period of adversity). Such individual adaptations have been termed allostatic resilience processes, as opposed to homeostatic resilience processes in which protective mechanisms are successfully engaged but an individual’s mode of operation in coping with adversity is not lastingly altered [12]. Malleable resilience factors are thus natural targets for prevention programs that aim to increase individual resilience [10, 15]. Studies have investigated several interventions designed to increase resilience, many of which focus on cognitive-behavioral or mindfulness-based methods, or a mix of both [16]. However, so far, many intervention studies to foster resilience present substantial methodological deficiencies such as missing a clear definition and operationalization of resilience, investigating effects of the intervention on single resilience factors instead of on outcome resilience, or the lack of baseline diagnostics or long-term follow-ups [17].

### The current study

The interventional study DynaM-INT of the EU Horizon 2020 project consortium DynaMORE (‘Dynamic Modelling of Resilience’ [18]) is designed to investigate two mobile- and wearable-based just-in-time adaptive interventions (JITAIs) aimed at fostering resilience and to predict their success based on participants’ baseline characteristics. The target sample consists of students and apprentices between 18 and 27 years. During this period of life, several mental disorders appear for the first time or even have their peak prevalence [19], and students seem to be a particularly vulnerable group for stress-related psychopathology [20–24]. Youth and emerging adults are also among the groups that were most strongly mentally affected by the COVID-19 pandemic [25]. Insofar as early-onset stress-related problems are often associated with life-long mental vulnerability, investment in the mental health of emerging adults is likely to yield lasting gains and to be economically particularly efficient [26]. To ensure that we specifically include at-risk individuals, inclusion criteria include the prior experience of at least three negative life events that are perceived as burdensome [27], and a score in the mid-to-high range of the 28-item version of the General Health Questionnaire (GHQ) [28], a self-report instrument that captures internalizing symptomatology.

**Fig. 1:**
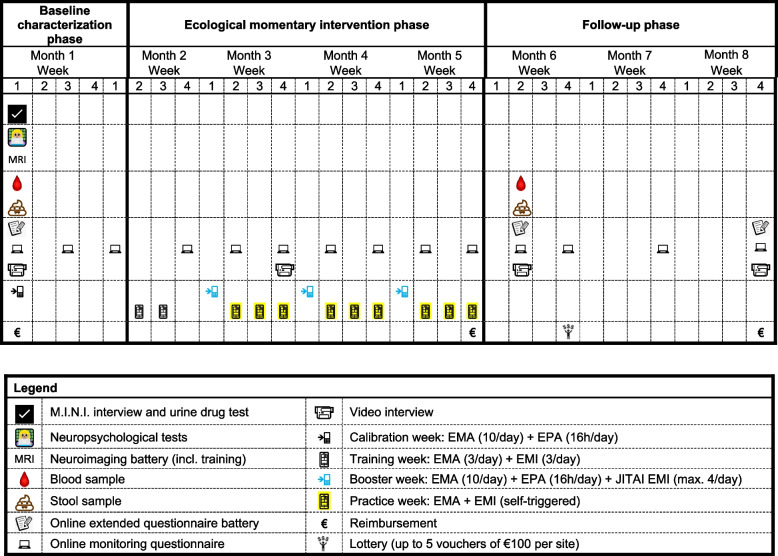
Study timeline. The study involves a baseline characterization phase, an ecological momentary intervention phase, and a follow-up phase. On-site assessments are done at the beginning of the baseline and follow-up phases. In Berlin, Tel Aviv, and Warsaw, all baseline on-site assessments are conducted on one day, while in Mainz and Nijmegen, these baseline assessments are split into two days: M.I.N.I. interview and blood sampling are done on day 1, all remaining procedures are performed on day 2. On both testing days in Mainz and Nijmegen, a urine drug test is conducted. On-site assessments are complemented by regular online monitoring of stressors, mental health problems, and selected resilience factors. Abbreviations: EMA, ecological momentary assessment; EMI, ecological momentary intervention; EPA, ecological physiological assessment; JITAI EMI, just-in-time ecological momentary intervention; M.I.N.I., Mini-International Neuropsychiatric Interview

As a prospective-longitudinal resilience study, DynaM-INT entails a multimodal baseline characterization phase that focuses on potential resilience factors followed by longitudinal, biweekly assessments of a small number of hypothesized key resilience factors, considered potentially malleable, as well as of experienced stressors (E) and mental health problems (P) throughout the course of the study (online monitoring questionnaires). See Fig. 1 for a schematic overview of the study timeline.

Repeated E and P monitoring implements the Frequent Stressor and Mental Health Monitoring (FRESHMO) paradigm, which we have developed specifically for the purpose of longitudinal resilience studies [12]. E and P scores are used to calculate stressor reactivity (SR) scores, the primary outcome variable and a proxy for outcome resilience [12] using a residualization approach [29, 30]. Specifically, we regress individuals’ mental health problems P on their stressor exposure E, both across all monitoring time points, to determine our sample’s normative E-P relationship. For any given individual timepoint, a participant’s regression residual from this normative E-P relationship reflects their SR relative to their current stressor exposure and the sample’s normative reactivity. Thus, positive residuals indicate that the participant experiences more mental health problems P than would be expected given their stressor exposure E (higher SR) at this time point, whereas negative residuals mean that a participant has fewer mental health problems than would be predicted at their given stressor exposure (lower SR) at this time point. Within-participant SR score time-courses will be calculated to investigate temporal fluctuations in reactivity and set these into relation with the interventions (see below) and with potential changes in resilience factors resulting from the interventions [12]. The repeated assessment of several potential resilience factors in the online monitoring questionnaires is complemented by repetitions of parts of the baseline characterization phase after six and eight months (‘follow-up phase’; Fig. 1).

Importantly, upon completion of the baseline characterization phase, participants enter an ecological momentary intervention (EMI) phase where they are randomly assigned to one of two EMIs designed by our consortium that aim to improve two distinct resilience factors: ‘ReApp’, targeting positive cognitive reappraisal of recent stressful or negative events [64], or ‘Imager’, targeting reward sensitivity by positive mental imagery [31, 65]. The interventions are accompanied by ecological momentary assessments (EMA) using smartphones and ecological physiological assessments (EPA) using wearables (wristbands) to assess mood and stress reaction patterns in real time during real life and to allow triggering of EMIs as JITAIs at times of high stress.

Specifically, after calibration of individual EMA and EPA thresholds for stress responses on study devices as part of baseline characterization (‘calibration week’, see Fig. 1), participants are first trained in using the assigned intervention on their own phones without concurrent EPA (‘training weeks’). Then, participants are administered three EMI ‘booster weeks’ on study devices during which real-time EMA and EPA data is used to trigger interventions specifically at moments when participants’ stress levels cross the individual threshold established during the calibration week (that is, JITAI). The rationale behind this approach is that these interventions are thought to be most effective when participants apply the previously learned cognitive strategies at moments when they are needed most [32]. These booster weeks happen every four weeks over the period of three months in the EMI phase. Between the booster weeks, participants are encouraged to continue practicing the assigned intervention (‘practice weeks’) on their own phones. Supplementary Figure S1 depicts the different assessments per week type [see Additional file 1].

### Research questions

The study is primarily designed to identify baseline predictors of the effect of our JITAIs on stressor reactivity as well as target engagement, in order to inform the design of future randomized controlled trials testing the efficacy of these interventions. To prepare predictor identification, we will first evaluate intervention feasibility and efficacy. We will evaluate feasibility by testing whether EMIs with a JITAI element that uses mobile phones and wristbands to trigger interventions specifically at times of high stress can be conducted on a large scale, focusing on i) technical implementation (feasibility research question 1, fQ1) as well as ii) participant adherence (fQ2) and iii) participant experience (fQ3).

To preliminarily evaluate the efficacy, we will quantify whether, relative to baseline, the interventions are accompanied by, i) reductions in SR scores (efficacy research question 1, eQ1) and ii) increases in respective target engagement (eQ2). For target engagement specifically, we will assess changes in the use frequency of positive cognitive reappraisal during and after the ReApp JITAI and changes in reward sensitivity during and after the Imager JITAI. These patterns could be interpreted as further evidence for intervention success [31, 64]. The efficacy tests primarily use the biweekly assessed self-report measures of stressor exposure, mental health, positive cognitive reappraisal, and reward sensitivity.

Our efficacy tests will be further facilitated by the possibility to compare DynaM-INT results to data from the purely observational DynaM-OBS study [33], to which DynaM-INT is the follow-up study. DynaM-OBS uses the same type of baseline characterization and repeated assessment of E, P, and resilience factors (specifically positive cognitive reappraisal) in a study sample and over a time period comparable to DynaM-INT. DynaM-OBS thus provides us with an estimate of the natural course of SR and target engagement measures that can be used as a discovery sample and as a background against which the effects of the interventions in DynaM-INT can be assessed. Note that DynaM-OBS cannot be considered a formal control condition, but may provide an informal effect estimate justifying future randomized controlled trials (RCTs) with appropriate control conditions.

Following these evaluations of feasibility (fQ1-3) and efficacy (eQ1-2), we will address our primary research questions, namely, examining variables assessed in the baseline characterization phase to identify those that moderate (predict) the efficacy of either of the two interventions on i) stressor reactivity (primary research question 1, pQ1) and ii) target engagement (pQ2). The exact list of potential moderator variables to be examined, besides initial levels of positive cognitive reappraisal and reward sensitivity, will depend on the results of the DynaM-OBS study. Specifically, in DynaM-INT we will focus on predictors of low SR scores obtained from DynaM-OBS. These investigations aim to prepare future RCTs intended to test the efficacy of these interventions where baseline data serves to guide intervention administration only to individuals that are likely to benefit from a given intervention.

As a follow-up to our two primary research questions, we will examine whether the anticipated reductions in stressor reactivity are preceded or accompanied by the anticipated increases in target engagement (secondary research question, sQ1), which would suggest that the interventions execute their effects via the targeted resilience mechanisms.

A tertiary set of main research questions (tertiary research question, tQ) to be answered with DynaM-INT is related to Positive Appraisal Style Theory of Resilience (PASTOR) [10], the core theoretical framework of the DynaM-INT study. Positive appraisal style (PAS) is the tendency of an individual to appraise potential stressors in a positive (i.e., non-negative) way while at the same time avoiding delusionally positive appraisals. Positive appraisers typically generate appraisals that range from realistic to slightly unrealistically positive. Such a positive appraisal style is thought to enable the individual to exhibit optimal, fine-tuned stress reactions that are sufficient to cope with the stressor but that do not exceedingly exhaust resources, which reduces the likelihood of developing mental health problems in adverse life situations. PASTOR claims that PAS is the key proximal resilience factor in that the effects of all other resilience factors on outcome resilience are mediated by their effects on PAS [10]. In PASTOR, positive cognitive reappraisal is one important sub-class of cognitive processes that generate positive appraisals [10, 34], and it is therefore claimed that individuals who use positive cognitive reappraisal more frequently and/or more efficiently are likely to have higher PAS. Thus, positive cognitive reappraisal is an important component of PAS, which is why it is here targeted by the ReApp EMI. By contrast, reward sensitivity, as targeted by the Imager EMI, is a separate potential resilience factor that is thought to promote resilience insofar as it helps individuals generally appraising stressful situations in a more benign fashion, by better integrating positive information into the overall appraisal. Hence, eventually, one can assume that both the ReApp and the Imager EMIs promote resilience by promoting PAS.

PAS (like positive cognitive reappraisal and reward sensitivity) is considered a malleable resilience factor. Accordingly, in our study design, self-report measures of PAS (like measures of the two EMI targets) are not only taken in the questionnaire battery of the baseline characterization phase but also when the characterization is repeated at follow-up as well as in the biweekly online monitoring questionnaires (see Fig. 1). This allows us to ask whether the interventions are accompanied by increases in PAS relative to baseline (tQ1), whether the anticipated reductions in stressor reactivity are preceded or accompanied by the anticipated increases in PAS (tQ2), and whether the anticipated increases in PAS are preceded or accompanied by the anticipated increases in target engagement (tQ3). These findings would suggest that the interventions promote resilience by promoting PAS. Beyond intervention effects, we will examine whether individuals with high baseline PAS show less stressor reactivity (tQ4) and whether changes in PAS throughout the course of the study will be accompanied by inverse changes in stressor reactivity (tQ5), irrespective of the treatment.

The research questions are summarized in Table 1; additional exploratory research questions are outlined in the analysis section. The DynaM-INT data set will be made available to researchers to address other possible research questions.

**Table 1:** List of research questions

Type	Nr	Research Question
Feasibility	fQ1	Is JITAI using mobile phones and wristbands to trigger interventions specifically at times of high stress technically feasible?
	fQ2	Do participants adhere to the JITAI?
	fQ3	How do participants experience the JITAI?
Efficacy	eQ1	Are the interventions accompanied by reductions in stressor reactivity relative to baseline?
	eQ2	Are the interventions accompanied by increases in target engagement relative to baseline?
Primary	pQ1	Can we identify predictors in the baseline characterization data for the effects of each of the two interventions on stressor reactivity?
	pQ2	Can we identify predictors in the baseline characterization data for the effects of each of the two interventions on target engagement?
Secondary	sQ1	Are the anticipated reductions in stressor reactivity preceded or accompanied by the anticipated increases in target engagement?
Tertiary	tQ1	Are interventions accompanied by increases in PAS relative to baseline?
	tQ2	Are the anticipated reductions in stressor reactivity preceded or accompanied by the anticipated increases in PAS?
	tQ3	Are the anticipated increases in PAS preceded or accompanied by the anticipated increases in target engagement?
	tQ4	Do individuals with high baseline PAS show less stressor reactivity?
	tQ5	Are changes in PAS throughout the course of the study accompanied by inverse changes in stressor reactivity?

In the DynaM-INT study, we attempt to answer multiple research questions, divided in feasibility and efficacy questions, as well as primary, secondary and tertiary main research questions

## Methods

### Study centers and study period

The multi-center study takes place in five research facilities: Department of Psychiatry and Neurosciences at Charité – Universitätsmedizin Berlin, Berlin, Germany; Neuroimaging Center at Johannes Gutenberg University Medical Center in Mainz, Germany; Donders Centre for Cognitive Neuroimaging and Radboud university medical center in Nijmegen, Netherlands; Sagol Brain Institute at Tel Aviv University and Tel Aviv Sourasky Medical Center, Tel Aviv, Israel, and Faculty of Psychology at University of Warsaw, Warsaw, Poland. Data acquisition started in April 2022. Completion of the baseline characterization phase is expected in May 2023, completion of the intervention phase is expected in September 2023, and completion of the follow-up phase is expected in December 2023.

### Participants

In total, *N* = 250 healthy male and female participants are planned to be recruited at the five study sites (*N* = 50 each). Where a study site cannot fulfil the recruitment goal, other sites will attempt to compensate. Participants need to be 18–27 years, studying or in vocational training, have experienced at least three stressful life events [27] that they perceived as burdensome before inclusion, and report elevated levels of internalizing symptoms (a score of ≥ 20 in the GHQ, 28-item version [28]). All inclusion criteria are provided in Table 2.

**Table 2:** List of inclusion criteria and format in which they were assessed

Nr	Criterion	Format
1	Age between 18 and 27	Online
2	3 or more life events rated as burdening [27]	Online
3	GHQ-28 score of 20 or higher [28]	Online
4	Body mass index between 18 and 27	Online
5	Currently studying or in vocational training	Online
6	Proficiency in the official language of the country of study enrollment (minimum level of C1 in the Common European Framework of Reference for Languages)	Online
7	Eligibility to participate in ecological physiological assessment using a wearable device (no skin disease in the wrist or chest area and no medical condition that increases risk of infection through electrodes, no medication with phototoxic side effects)	Online
8	The participant has a smartphone with iOS or Android operating system	Online
9	No lifetime diagnosis of any severe mental or organic disorder that affects neurodevelopment due to its pathological mechanism or treatment (e.g., schizophrenia, bipolar disorder, anorexia/bulimia nervosa, attention deficit hyperactivity disorder, autism spectrum disorder, meningitis, epilepsy, multiple sclerosis, stroke, brain cancer, brain concussion, or coma)	Online + interview
10	Eligibility for undergoing the functional magnetic resonance imaging protocol (normal or corrected-to-normal eyesight, no hearing impairment, no claustrophobia, no non-removable ferromagnetic metal in or at the body, not pregnant, no large tattoo in head or neck area)	Online + interview
11	No diagnosis within 9 months before inclusion of any mental disorder other than a mild depressive episode (ICD F32.1), tobacco abuse/dependence (ICD F12), or substance abuse, as assessed using the Mini-International Neuropsychiatric Interview (M.I.N.I.)[36]	Interview
12	The participant has not participated in the previous DynaM-OBS study or any study using an EMI similar to ReApp or Imager	Interview
13	No consumption of any psychoactive drug or substance up to 4 weeks prior to the first psychological assessment and to the MRI assessment	Drug test
14	The participant has received all relevant information about the study, is able to obtain full insight and is fully contractually capable, is willing and able to comply with the protocol and agrees to participate by giving written consent	Interview

Participants who are found eligible in criteria 1–10 in the anonymous online screening are invited to a phone interview (on-site interview in Mainz and Nijmegen) to confirm/check eligibility for criteria 9–12. During their first in-person appointment, participants receive written and verbal information about the study and provide written informed consent (criterion 14). Inclusion criterion 10 only applies to the MRI subsample: participants who are not eligible for undergoing the MRI procedure skip the neuroimaging procedures and take part in all other parts of the study. During the baseline day (both baseline days in the case of Mainz and Nijmegen see Fig. 1), a drug test is performed

### Design

As shown in Fig. 1, the DynaM-INT study follows a prospective-longitudinal design, consisting of an (online) pre-screening for eligibility, a multimodal baseline characterization phase (including neuropsychological tests, neuroimaging, bio-samples, a sociodemographic and psychological questionnaire battery, a video-recorded interview), a calibration week where individual stress thresholds are being determined based on ecological momentary assessments (EMA) and ecological physiological assessments (EPA)), an ecological momentary intervention (EMI) phase (including two training weeks where participants get familiar with one of two randomly assigned interventions, three separated booster weeks where JITAIs are triggered at times of high stress, intermittent optional EMI practice weeks without JITAI, and another video-recorded interview), and a follow-up phase where parts of the baseline characterization phase are repeated (including the psychological questionnaire battery, bio-samples, and the video-recorded interview). In addition, biweekly online monitoring questionnaires are assessed throughout the course of the study. For an extensive overview of all measures used and the days (d), weeks (w) and months (M) from baseline at which they are assessed (x), see Table 3.

**Table 3:** Overview of the measures used and the weeks (w) and months (M) from baseline, at which they are assessed (x: all sites; a: Mainz and Nijmegen; b: Berlin, Tel Aviv, and Warsaw)

			**S**	**Baseline** **characterization phase**							**Ecological momentary intervention phase**															**Follow-up phase**											
				**M1**					**M2**					**M3**				**M4**				**M5**				**M6**				**M7**				**M8**			
				**w0**	**w1**	**w2**	**w3**	**w4**	**w1**	**w2**		**w3**	**w4**	**w1**	**w2**	**w3**	**w4**	**w1**	**w2**	**w3**	**w4**	**w1**	**w2**	**w3**	**w4**	**w1**	**w2**	**w3**	**w4**	**w1**	**w2**	**w3**	**w4**	**w1**	**w2**	**w3**	**w4**
**Inclusion**	Inclusion criteria		**x**																																		
	M.I.N.I. Interview		**x**																																		
	Drug screening			**a**	**x**																																
**Mental health**	**Online Questionnaires**																																				
	GHQ-28	General Health Questionnaire	**x**		**x**		**x**		**x**				**x**		**x**		**x**		**x**		**x**		**x**		**x**		**x**		**x**				**x**				**x**
	PSS-10	Perceived Stress Scale			**x**																						**x**										**x**
	SCL-90-R	Symptom Checklist 90 Revised			**x**																						**x**										**x**
	WHO-DAS	WHO Disability Assessment Scale			**x**																						**x**										**x**
	DBM	Digital Biomarkers			**x**												**x**										**x**										**x**
**Stressor Exposure**	**Online Questionnaires**																																				
	MIMIS	Mainz Inventory of Micro-stressors			**x**		**x**		**x**				**x**		**x**		**x**		**x**		**x**		**x**		**x**		**x**		**x**				**x**				**x**
	COV Stress	Covid-related Stressors			**x**		**x**		**x**				**x**		**x**		**x**		**x**		**x**		**x**		**x**		**x**		**x**				**x**				**x**
	War and Terror Stress	War- and Terror-related Stressors			**x**		**x**		**x**				**x**		**x**		**x**		**x**		**x**		**x**		**x**		**x**		**x**				**x**				**x**
	LEQ	Life Event Questionnaire	**x**		**x**																						**x**										**x**
**Potential Resilience** **and Risk Factors**	**Neuropsychological battery**				**x**																																
	**Neuroimaging battery**				**x**																																
	**Online Questionnaires**																																				
	PASS-content	Perceived Positive Appraisal Style Scale, content-focused			**x**^**S**^		**x**^**M**^		**x**^**M**^				**x**^**M**^		**x**^**M**^		**x**^**M**^		**x**^**M**^		**x**^**M**^		**x**^**M**^		**x**^**M**^		**x**^**S**^		**x**^**M**^				**x**^**M**^				**x**^**S**^
	PASS-process	Perceived Positive Appraisal Style Scale, process-focused			**x**^**S**^		**x**^**M**^		**x**^**M**^				**x**^**M**^		**x**^**M**^		**x**^**M**^		**x**^**M**^		**x**^**M**^		**x**^**M**^		**x**^**M**^		**x**^**S**^		**x**^**M**^				**x**^**M**^				**x**^**S**^
	TEPS	Temporal Experiences of Pleasure			**x**^**S**^		**x**^**M**^		**x**^**M**^				**x**^**M**^		**x**^**M**^		**x**^**M**^		**x**^**M**^		**x**^**M**^		**x**^**M**^		**x**^**M**^		**x**^**S**^		**x**^**M**^				**x**^**M**^				**x**^**S**^
	CPA	Crisis-related Positive Appraisals			**x**		**x**		**x**				**x**		**x**		**x**		**x**		**x**		**x**		**x**		**x**		**x**				**x**				**x**
	Other primary resilience and risk factors				**x**																						**x**										**x**
	Secondary resilience and risk factors				**x**																						**x**										**x**
	Sports and mental activities				**x**																						**x**										**x**
	**Bio-samples**																																				
	EDTA Blood	DNA/DNA-methylation		**a**	**b**																						**x**										
		Proteomics		**a**																																	
	Stool	Microbiome			**x**																						**x**										
**Daily life measures**	**Ambulatory Assessments**																																				
	EPA	Ecological Physiological Assessment				**x**								**x**				**x**				**x**															
	EMA	Ecological Momentary Assessment				**x**				x*		x*		**x**	**x**^**#**^	**x**^**#**^	**x**^**#**^	**x**	**x**^**#**^	**x**^**#**^	**x**^**#**^	**x**	**x**^**#**^	**x**^**#**^	**x**^**#**^												
	EMI	Ecological Momentary Interventions								x*		x*			**x**^**#**^	**x**^**#**^	**x**^**#**^		**x**^**#**^	**x**^**#**^	**x**^**#**^		**x**^**#**^	**x**^**#**^	**x**^**#**^												
	JITA IEMI	Just-in-time Ecological Momentary Interventions												**x**				**x**				**x**															
	**Online Questionnaire**																																				
	uMARS	User version of the Mobile Application Rating Scale questionnaire																									**x**										

Resilience and risk factors (RFs) are grouped into primary and secondary RFs. Primary RFs are of main interest in the current study based on previous findings and theoretical background of our consortium, while secondary RFs are based on hypotheses drawn from the literature. RFs addressing target engagement (assessed with a subset of items in the PASS-process and the TEPS questionnaire) are assessed as traits or styles (typical tendencies, habits, x^S^) during the extended online batteries in M1, M6, and M8 and as modes (response tendencies in the past days or weeks, x^M^) in the repeated online monitoring questionnaires. During training (x*) and practice weeks (x^#^), EMIs are preceded by an EMA. During practice weeks (x^#^), EMIs are self-triggered/on-demand

Abbreviations: *M* month, *S *screening phase, *w* week

### Procedures

#### Recruitment and screening

Participants are recruited via e-mail distribution lists, social media advertisements, flyers, digital blackboards, and word-of-mouth. As a first step, potential participants are asked to fill out an anonymous online screening survey on SoSci Survey [36] that checks for inclusion criteria (Table 2) via an automated algorithm. To be able to link the pre-screening data to the study ID, potential participants generate an individual code that will be re-created on-site upon inclusion. Eligible participants receive an e-mail with the invitation to contact their study site to schedule a phone call.

Further inclusion criteria regarding past and present psychiatric diagnoses are assessed by trained staff using the Mini-International Neuropsychiatric Interview (M.I.N.I.) [35]. In Berlin, Tel Aviv, and Warsaw, the M.I.N.I. is conducted on the phone and all records are destroyed afterwards. Eligible participants are then scheduled for the baseline characterization phase. In Mainz and Nijmegen, participants receive an appointment for the first day of baseline assessments during which the M.I.N.I. is conducted and participants who are not eligible are treated as dropouts.

#### Baseline characterization phase (month 1)

Participants are characterized in a multimodal baseline characterization phase, consisting of on-site assessments, as well as online questionnaires and assessments in daily life. An overview of all procedural steps of the baseline assessments can be found in Table 4.

**Table 4:** Procedure steps at baseline

	**Procedure step**	**Task/sample**	**Self-ratings**	**Duration (mm:ss)**
	Phone screening	M.I.N.I. interview (Berlin, Tel Aviv, & Warsaw)		
Day 1		Informed consent (Mainz & Nijmegen)		
	On-site screening	M.I.N.I. interview (Mainz & Nijmegen)		
		Drug screen (Mainz & Nijmegen)		
	Bio-samples	Blood (Mainz & Nijmegen)		
	Post-assessment	Longitudinal schedule (Mainz & Nijmegen)		
		Online questionnaire briefing and DBM training (Mainz)		
		Emotional disturbances interview (Mainz & Nijmegen)		
Day 2	Pre-neuroimaging	Drug screen		
		Informed consent (Berlin, Tel Aviv, & Warsaw)		
		M.I.N.I. interview (Berlin, Tel Aviv, & Warsaw)		
		Neuroimaging training		
	Neuropsychology	Trail making test		01:30
		Digit symbol test		01:30
	Bio-samples	Blood (Berlin, Tel Aviv, & Warsaw)		
		Stool instruction		
	Neuroimaging battery	Reward sensitivity task (MID)		08:26
		T1		06:54
		Reappraisal task	Performance	13:06
		Faces matching task		04:34
		FLAIR		02:44
		Resting state		07:10
	Post-neuroimaging	MRI exit interview		
		EMA/EPA briefing		
		Online questionnaire briefing and DBM training (Berlin, Nijmegen, Tel Aviv & Warsaw)		
		Longitudinal schedule (Berlin, Tel Aviv, & Warsaw)		
		Emotional Disturbances Interview		
Day 3–8	Calibration week	EMA/EPA data collection		

Note that the M.I.N.I. interview is conducted twice in Berlin, Tel Aviv, and Warsaw because all records collected previous to informed consent only serve the purpose of checking inclusion criteria and are immediately destroyed. Before each neuroimaging sequence, a field map scan is acquired. The total duration of the imaging battery is about 1 h. Abbreviations: EMA, ecological momentary assessment; EPA, ecological physiology assessment; FLAIR—fluid-attenuated inversion recovery; M.I.N.I., Mini-International Neuropsychiatric Interview; T1, T1- weighted image

In Berlin, Tel Aviv, and Warsaw, all on-site baseline assessments are conducted on one day (“day 1 + day 2” in Table 4). In Mainz and Nijmegen, on-site baseline assessments are split into two days: In Nijmegen, the M.I.N.I., and blood sampling are done on day 1; in Mainz, the M.I.N.I., blood sampling, and EMA/EPA briefing are done on day 1. All remaining on-site assessments are performed on day 2. In Berlin, Tel Aviv, and Warsaw, participants spend approximately 4 h in the laboratory during day 1. In Mainz and Nijmegen, they are present for approximately 1 and 3 h(s) on day 1 and 2, respectively.

All participants receive written and verbal information about the study and provide written informed consent at the start of the baseline assessment. Next, (at the start of both baseline days in the case of Mainz and Nijmegen), participants undergo a urine-based drug screening test (SureStep™ Multi-Drug One Step Screen Test Panel, Innovacon Inc., USA) for amphetamine, barbiturates, benzodiazepines, buprenorphine, clonazepam, cocaine, fentanyl, heroin, ketamine, cannabis, methadone, methamphetamine, methylenedioxymethamphetamine, morphine, opiate oxycodone, phencyclidine, propoxyphene, tramadol, and tricyclic antidepressants. After a negative test, participants continued with the tests.

##### Neuropsychological tests

Following inclusion, two neuropsychological tests are conducted: the Trail Making Test [37, 38], assessing visual attention and task switching speed, and the HAWIE Digit Symbol Test [39], measuring processing speed.

##### Neuroimaging

Participants receive a brief training of the neuroimaging paradigms, during which the experimenter provides verbal explanations, asks questions, and makes sure the participant understood the instructions, while showing an on-screen presentation of the tasks. Data acquisition parameters and the individual neuroimaging tasks are described in detail below.

When placed in the magnetic resonance imaging (MRI) scanner, participants are provided with earplugs. They receive a 4-button Inline Fiber Optic Response Pad (Current Design [40], in Berlin, Mainz, Nijmegen, and Tel Aviv; in-house developed system in Warsaw) to their right hand. They are presented with the visual stimulation of the tasks via a mirror placed on the head coil that shows a monitor placed behind the scanner bore. Before and after each task, the experimenter gives verbal instructions and receives feedback from the participant via an intercom system. The specific instructions are also shown on the screen before each task. After scanning, participants are asked to fill out an MRI exit interview questionnaire, asking about experiences and potential difficulties with the fMRI tasks, via SoSci Survey [36].

Participants who are not eligible for undergoing the MRI procedure skip the neuroimaging procedures and take part in all other parts of the study.

##### Bio-samples

From each participant, 9 ml of blood (in Nijmegen: 10 ml) is drawn into an EDTA tube (red monovette; Sarstedt, Nümbrecht, Germany) and stored as whole-blood at -20 °C or colder until assay of DNA and DNA-methylation. In Mainz and Nijmegen, an additional 9 ml (Mainz) or 10 ml (Nijmegen) of blood are sampled into EDTA tubes for proteomic analyses. To limit the influence of metabolism or diurnal oscillations on proteomics measurements, at these two sites blood is drawn between 10:30 and 14:30 and participants are instructed to arrive at least five hours sober. Blood samples for proteomics assay are centrifuged and serum is divided into 8–16 aliquots (depending on volume), which are stored at -80 °C until assay. In Tel Aviv, one additional tube (VACUETTE® TUBE 5 ml CAT Serum Separator Clot Activator) of blood is taken at each sampling time point to derive CRP.

Stool samples are collected using an OMNIgene-gut feces kit (OM-200, DNAgenotek). Participants receive a test kit, an instruction sheet about the collection procedure, the Bristol Stool Scale [41], and a verbal instruction. They are instructed to collect the stool sample as close as possible to the return appointment, to take numerous small samples from different locations in the stool material, to fill out the Bristol Stool Scale, and to store the sample at a dark place without direct sunlight until returning it at the next appointment. Stool samples are subsequently stored at -20 °C until assay of gut microbiome, or, in Nijmegen, directly shipped to the laboratory processing the microbiome.

##### Post-assessment procedures

At the end of the baseline day(s) (and each subsequent appointment), participants are asked if they have experienced emotional disturbances triggered by any element of the preceding session in a standardized interview, to ensure their well-being. In case they report emotional disturbance and a need for help, participants are directed to a site-specific clinician associated with the study.

##### Online questionnaires

Following the on-site baseline day (Mainz and Nijmegen: baseline day 2), a schedule with the participant’s dates for all questionnaires is uploaded to SoSci Survery [36] to enable automatic e-mail dispatch. The schedule consists of an extended questionnaire battery, as well as shorter, biweekly monitoring questionnaires, used for the high-frequent longitudinal assessment of stressors and mental health (FRESHMO paradigm) as well as of malleable resilience factors (RFs) throughout the entire study [12]. RFs are assessed as trait or style (the typical way or tendency in which a person reacts to life experiences) during the extended online batteries and as a mode (the extent to which the RF was used or experienced in the past two weeks [42]) during the biweekly monitoring questionnaires. Table 5 provides an overview.

**Table 5:** List of online self-report questionnaires

Type	Name (Abbreviation)	Construct
**Mental health**		
	General Health Questionnaire (GHQ-28)	Symptoms of anxiety, depression, insomnia, social problems as well as somatic symptoms. This inventory is designed to capture the inability to carry out normal functions and the appearance of new and distressing phenomena in the general population, 28 items [28]
	Perceived Stress Scale (PSS-10)	Degree to which participants appraise situations in their lives as stressful, unpredictable, uncontrollable, and overloaded, 10 items [70, 71]
	Revised Symptom Checklist 90 (SCL-90-R)	Psychological distress in terms of nine primary symptom dimensions including somatization, obsessive–compulsive, interpersonal sensitivity, depression, anxiety, hostility, phobic anxiety, paranoid ideation, and psychoticism, 90 items [72]
	WHO Disability Assessment Schedule (WHODAS 2.0)	Functioning and disability in accordance with the International Classification of Functioning, Disability and Health, 12 items [73]
**Stressor exposure**		
	Mainz Inventory of Microstressors (MIMIS)	58 minor stressors of daily life (e.g., loss or displacement of an object, interpersonal conflicts, bad weather, traffic). Participants report whether the events have occurred and how straining they were experienced on a 5-point scale [44]
	List of COVID-related stressors (COV stress)	A list of 23 stressors specific to the COVID-pandemic (e.g., being at increased risk for an infection, loss of social contact, having COVID symptoms, etc.), for which participants report whether the situation occurred and how burdensome it was perceived on a 5-point scale. The list was self-developed in March 2020 for the DynaCORE studies on psychological resilience during the COVID-pandemic [42, 74]
	List of War- and Terror-Related Stressors	A self-developed list of 15 stressors related to the war in Ukraine, for which participants report whether the situation occurred and how burdensome it was perceived on a 5-point scale [63] and a self-developed list of 5 terror terror-related stressors for which participants report whether they experienced the situation [63]
	Life Events Questionnaire (LEQ)	27 stressful life events (e.g., death of a friend or family member, separation or divorce of the parents, illness or injury). For each event, participants indicate whether and at what age it has occurred and how positive or burdensome it has been experienced [27]
**Questionnaires used to assess primary resilience and risk factors**		
	Perceived Positive Appraisal Style Scale, content-focused (PASS-content)	Assessment of the perceived tendency to generate positive appraisals in challenging situations. The instrument has 14 items; answers are given on a 4-point scale [45]
	Perceived Positive Appraisal Style Scale, process-focused (PASS-process)	Assessment of the perceived tendency to employ positive appraisal processes in challenging situations. The instrument has 10 items; answers are given on a 5-point scale [45]
	Temporal Experience of Pleasure (TEPS)	Reward sensitivity is assessed using four anticipatory items from the TEPS [75]
	List of crisis-related positive appraisals	A list of 6 self-formulated positive appraisal contents specific to the current crisis [63]
	Brief Resilience Scale (BRS)	The subjective ability to cope with and recover from stress, 10 items [76]
	Cognitive Emotion Regulation Questionnaire (CERQ short)	Different strategies of emotion regulation such as self-blame, other-blame, rumination, catastrophizing, positive refocusing, planning, positive reappraisal, putting into perspective, and acceptance, 18 items [90]
	Coping Orientation to Problems Experienced questionnaire (Brief COPE)	Emotion regulation strategies such as self-distraction, active coping, denial, substance use, use of emotional support, use of instrumental support, behavioral disengagement, venting, positive reframing, planning, humor, acceptance, religion, and self-blame, 28 items [77]
	General Self Efficacy Scale (GSE)	Perceived ability to cope with a variety of difficult demands in life, 10 items [46]
	Internal External Locus of Control-4 (IE-4)	Degree to which individuals perceive themselves the outcomes of their behavior to be determined by their own actions or by forces outside of their control 4 items, 4 items [47]
	Life Orientation Test – Revised (LOT-R)	Dispositional optimism and pessimism, 10 items [48]
	NEO-Neuroticism	Neuroticism scale of the NEO Five Factor Inventory (NEO-FFI), 12 items [78]
	Oslo 3 Item Social Support Scale (OSSS-3)	Degree to which participants perceive themselves as surrounded by people who are close, concerned, and supportive [79]
	Psychological Flexibility Questionnaire (PFQ)	Subjective psychological flexibility, assessed via five factors including positive perception of change, characterization of the self as flexible, self-characterization as open and innovative, a perception of reality as dynamic and changing, and a perception of reality as multifaceted, 20 items [80]
**Questionnaires used to assess secondary resilience and risk factors**		
	Anxiety Sensitivity Index (ASI)	Beliefs of negative implications of anxiety experiences, 18 items [81]
	Dimensional Anhedonia Rating Scale (DARS)	Multiple facets of hedonic function such as desire, motivation, effort, and consummatory pleasure across hedonic domains, 17 items [82]
	Maltreatment and Abuse Chronology of Exposure (MACE)	Abuse and neglect during development, 52 items [83]
	Perceived Social Status Scale (PSS-S)	Subjective socioeconomic status by means of a drawing of a ladder with 10 rungs, described to represent where people stand in society. Participants are instructed to indicate the rung that best represents where they stand on the ladder. Additionally, the same question is asked for the dimensions of academic and occupational status [84]
	Ruminative Thought Style Questionnaire (RTS)	Components of ruminative thinking including problem-focused thoughts, counterfactual thinking, repetitive thoughts, and anticipatory thoughts, 15 items [85]
	Sensitivity to Punishment and Sensitivity to Reward Questionnaire (SPSRQ)	Tendency for aversive and appetitive behavior, 48 items [86]
	State Trait Anxiety Inventory (STAI)	Symptoms of anxiety as a state and as a general trait, respectively, 40 items [87]
	Toronto Alexithymia Scale (TAS-20)	Deficiency in understanding, processing, or describing emotions, 20 items [88]
**Questionnaire used to assess user experience of the intervention**		
	User version of the Mobile Application Rating Scale (uMARS)	A list of 10 questions, adapted from the user version of the user version of the Mobile Application Rating Scale (uMARS) questionnaire [89]

Resilience and risk factors (RFs) are grouped into primary and secondary RFs. Primary RFs are of main interest in the current study based on previous findings and theoretical background of our consortium, while secondary RFs are based on hypotheses drawn from the literature. Please note that only the original publications are cited here but not the validation studies of translated versions into the four study languages. An overview of questionnaire validations, as well as the self-developed questionnaires are provided at [63]

The extended questionnaire battery is administered as part of the baseline characterization phase and is sent out immediately. Participants are asked to finish the online questionnaire battery within one week. Also, three biweekly monitoring questionnaires form part of the baseline characterization phase (see Fig. 1). Participants have two days to fill out those shorter questionnaires.

##### Video-recorded interview

Besides traditional self-report instruments, the online questionnaire schedule contains a self-developed, fully structured and video-recorded interview asking participants about their experience of mental health problems as well as recent and upcoming emotional events. In each interview, participants record short video segments of themselves answering the respective questions. These interviews provide audio-visual data to identify interview-based digital biomarkers of mental health (DBMs). Details are given below.

##### Calibration week

In the week following the on-site baseline assessment day(s), EMA and EPA data is collected. Participants use a study smartphone (Motorola Moto E6 Play in Berlin, Mainz, Nijmegen, and Warsaw; Xiaomi Redmi 7/7A in Tel Aviv) with the RADAR aRMT app (adapted for the use in DynaM-INT) for EMA data collection [51] and the Chill + wristband (developed by IMEC [52]) for EPA data collection. Participants receive a thorough explanation about the EMA and EPA devices, applications, and procedures.

Each day during usual waking hours (between 7:30 and 22:30), questionnaires of around 2 min length each are sent at 10 different time points (“beeps”) via push notifications to the smartphone. Each notification is semi-randomly scheduled to be sent out in a block of 90 min. The beep schedule is the same for all participants and is specified in Supplementary Table S1; EMA content can be found in Supplementary Figure S2 [see Additional file 1]. Each beep questionnaire remains online for 10 min, and participants receive a reminder notification 5 min after the initial beep notification.

EPA data is collected via the wristband for 16 h per day. The wristband also features a “stress” button that participants are instructed to press when they experience a stressful event. The calibration week lasts for six days.

All EMA data collected with the RADAR aRMT app is immediately and automatically uploaded to a server at the Donders Institute, where the initial feature extraction takes place in real time. After completion of each EMA questionnaire (via the RADAR aRMT app) participants are redirected to the DynaMORE Chill + app (developed by IMEC for the use in DynaM-INT) to upload 10 min of EPA data acquired right before each EMA notification to the server at the Donders Institute, where relevant features are extracted and motion-related artifacts are removed. A complete list of features is given in Supplementary Table S2 [see Additional file 1].

After the calibration week has finished, participants come back to the lab to return study devices. All data collected with the Chill + app is downloaded by the researchers for additional offline feature extraction (of the entire 6 days × 16 h EPA data). The baseline characterization phase is completed by randomly assigning one of the interventions to the participant based on a predetermined randomization sheet (computerized random numbers to 1 of 2 EMIs).

#### Ecological momentary intervention phase (months 2–5)

The ecological momentary intervention (EMI) phase consists of two training weeks, three booster weeks, and nine encouraged practice weeks. See Fig. 1. Also, online monitoring questionnaires continue to be sent to participants in a biweekly manner throughout the entire EMI phase. The video-recorded interview is repeated during the month 3—week 4 monitoring questionnaire.

##### Training weeks

Before the start of the two training weeks (14 days), participants receive a briefing on their assigned intervention (ReApp or Imager EMI) via a video call. Subsequently, they install the SEMA3 app [49] on their own phone and enroll for the assigned EMI. The purpose of the training weeks is to familiarize the participants with the assigned intervention and to initiate habitual use of the cognitive techniques taught by the app. Participants receive three daily EMIs via push notifications, scheduled throughout the day during pseudo-random one-hour time windows (at 10:00, 14:30, and 19:00). Participants have 20 min to execute the EMI after they receive the push notification. Researchers are automatically notified by mail if compliance drops below 60%. In that case, participants are contacted to resolve potential problems. In addition, participants are asked to complete one EMI before going to bed (on demand). Participants are encouraged to manually start (additional) interventions whenever they want to. EMIs are always preceded by an EMA, which is identical to the EMAs performed during calibration. EMI and EMA content is given in the SEMA3 app during the training weeks.

##### Booster weeks

Before the start of the first booster week, participants receive a refresher briefing, either in person when they pick up their devices, or via a video call. During the booster weeks, EMA and EPA data are collected analogously to the calibration week, using the RADAR aRMT app on study smartphones and Chill + wristbands. Incoming EMA and EPA data are analyzed in real time on a high-performance computing cluster at the Donders Centre for Cognitive Neuroimaging in Nijmegen. If the combination of extracted features exceeds the individual threshold (set to a goal of triggering three interventions per day, based on stressful situations from the calibration week), the assigned intervention is immediately triggered via the RADAR-BASE platform. The intervention arrives ~ 20 min after the start of the EMA questionnaires. A maximum of four interventions are triggered per day. Thresholds are adjusted on a daily basis to accommodate signal drift.

Additionally, each day starts with a morning questionnaire and ends with an evening questionnaire also shown in the RADAR aRMT app on the study smartphone, given in Supplementary Table S2 [see Additional file 1]. The evening questionnaire is followed by an additional intervention, ensuring that all participants receive at least one intervention per day. Participants are encouraged to start additional interventions themselves whenever they want to. Each booster week lasts for six days.

##### Practice weeks

Participants are encouraged to use the SEMA3 app on their own phone during the remaining weeks of the EMI phase (i.e., during the weeks in between booster weeks). During these encouraged practice weeks, participants do not receive notifications but are instructed to complete EMIs whenever they want to. Again, EMIs are always preceded by an EMA.

#### Follow-up phase (months 6–8)

Online monitoring continues during the follow-up phase and changes from biweekly to once a month during months 7 and 8. The extended online questionnaire battery is repeated during month 6—week 2 and month 8—week 4. Both assessments also include the video-recorded interview. In month 6—week 2, user experience of the JITAI is assessed with an adapted version of the user version of the Mobile Application Rating Scale (uMARS) questionnaire [53]. Follow-up blood and stool samples are also collected in month 6—week 2. See Fig. 1.

#### Remuneration

Complete participation in all assessments is remunerated with 340 EUR (in Tel Aviv 1200 NIS, in Warsaw 1200 PLN). Further, participants can win on average 10 EUR (40 NIS, 40 PLN) during the Monetary Incentive Delay task in the neuroimaging battery. Participants who finish all assessments are additionally included in a lottery to win a 100 EUR / 400 NIS / 400 PLN voucher on top (five vouchers in Berlin, Mainz, Nijmegen, and Tel Aviv; one in Warsaw). To maintain compliance throughout the longitudinal assessments, money is disbursed in tranches at different time points throughout the study, depicted in Supplementary Table S3 [see Additional file 1].

### Materials

#### Neuroimaging

##### MRI data acquisition

In Berlin, Mainz, Nijmegen, and Tel Aviv, brain imaging data are acquired on identical models of 3 T MAGNETOM Prisma systems (Siemens Healthineers, Erlangen, Germany) with 32-channel head coils (Tel Aviv: 64-channel head coil) using the following settings: Multiband gradient-echo echo planar imaging (EPI) sequences (TR = 800 ms, TE = 37 ms, flip angle = 52°, FOV = 208 mm, voxel size = 2.0 × 2.0 × 2.0 mm, 72 slices, MB acceleration factor = 8, phase-encoding direction = PA) from the Center for Magnetic Resonance Research, University of Minnesota, as adopted from the Human Connectome Project, are used for blood oxygen-level dependent (BOLD) fMRI [53]. Before each task, a pair of blip-up/blip-down EPI sequences is acquired (TR = 8000 ms, TE = 66 ms, flip angle = 90°, FOV = 208 mm, voxel size = 2.0 × 2.0 × 2.0 mm), one with an AP and one with a PA phase-encoding direction. Furthermore, a T1-MPRAGE sequence (TR = 2500 ms, TE = 2.22 ms, flip angle = 8°, FOV = 256 mm, voxel size = 0.8 × 0.8 × 0.8 mm) and a FLAIR sequence (TR = 9000 ms, TE = 83 ms, flip angle = 150°, FOV = 220 mm, voxel size = 0.7 × 0.7 × 3.0 mm) are acquired.

In Warsaw, a 3 T MAGNETO Trio system (Siemens, Germany) is used until October 2022. There, multiband gradient-echo EPI sequences are acquired with the following settings: TR = 1410 ms, TE = 30.4 ms, flip angle = 56°, FOV = 210 mm, voxel size = 2.5 × 2.5 × 2.5 mm, 60 slices, MB acceleration factor = 3, phase-encoding direction = PA. Additionally, blip-up/blip-down EPI sequences before each task (identical settings as other sites, except for voxel size = 2.5 × 2.5 × 2.5 mm), a T1-MPRAGE (TR = 1100 ms, TE = 3.32 ms, flip angle = 7°, FOV = 256 mm, voxel size = 1.0 × 1.0 × 1.0 mm), and a FLAIR sequence with identical settings as above are acquired. In October 2022, Warsaw replaced the Trio system with a 3 T MAGNETOM Prisma system (Siemens Healthineers, Erlangen, Germany) with 32-channel head coils using the same settings as Berlin, Mainz, Nijmegen, and Tel Aviv (described above).

Head movement is restricted by foam pads and tape on the forehead. All task paradigms are presented using the software Presentation® (Neurobehavioral systems [54]) on a monitor placed behind the scanner bore via a mirror that is fixed on the head coil.

##### Reward sensitivity task

An adapted version of the Monetary Incentive Delay Task (MID) [55] is used to measure neural responses during anticipation and receipt of rewards and losses [56]. Participants are told that they can win or lose a small amount of money if they press a button fast enough once a target stimulus (white star) appears on the screen. Right before the target appears, a cue that is presented for 2 s indicates whether they can win (+ 3€/12NIS/12PLN, + 0.5€/2NIS/2PLN), lose (-0.5€/2NIS/2PLN, -3€/12NIS/12PLN) or neither win nor lose (0€/NIS/PLN) money during the following trial. The cue is followed by a jittered anticipation phase of 2–2.5 s, after which participants need to press a button with their index finger as soon as the target stimulus appears on the screen. Each trial ends with a 2 s numeric feedback on subjects’ trial outcome as well as the overall gain. An adaptive algorithm is applied that changes the duration of target presentation for the participant within each condition based on their past performance to ensure that the experience of reward does not differ between subjects depending on their task performance. If the participant’s hit rate is below 66%, the target duration is increased by 25 ms; else, it is reduced by 25 ms. Reaction times and hit rates are collected as behavioral outcomes. A graphical depiction of the task design is provided in Supplementary Figure S3 [see Additional file 1]. The reward sensitivity task was used identically in the DynaM-OBS study [33] and the Mainz Resilience Project (MARP) study [56, 57].

Note that the DynaM-OBS data set will be used to identify the reward-related behavioral and neural measures from the task that are prospectively most strongly negatively associated with participants’ SR scores during that study [33]. These will be used in DynaM-INT as baseline indices of the targeted resilience factor reward sensitivity, complementary to the questionnaire-based self-report measures (see below). They will be tested in the main analyses of DynaM-INT as potential moderators of intervention effects (primary research questions on intervention success prediction, see Introduction and Table 1).

##### Situation-focused volitional reappraisal task

In the situation-focused volitional reappraisal task, assessing the ability to use positive cognitive reappraisal (reappraisal efficacy, reappraisal performance), participants are instructed to positively reinterpret or just view photographs which are either negative, positive, or neutral and to subsequently rate their affective state on a non-verbal scale [56, 58]. Stimuli were selected from the International Affective Picture System (IAPS) [59] and EmoPics [60] based on normative ratings regarding valence and arousal. For details on the task design, see Supplementary Figure S4 [see Additional file 1]. The situation-focused volitional reappraisal task was used identically in the DynaM-OBS study [33]. Timing of the current task is identical to the MARP study [56, 57], but a different set of IAPS/EmoPics stimuli [59, 60] is used.

Note that the same approach as above for the reward sensitivity task will be used to decide which measures from this task to include in the main analyses of DynaM-INT.

##### Implicit emotion processing task

An adaptation of the face matching task [61, 62] is used to assess the participants’ neural responses during implicit emotion processing. In each trial, participants are presented with one picture at the top and two pictures at the bottom part of the screen, of which one is identical to the upper one. They are instructed to select the matching picture from the bottom row by pressing a button. In the emotion condition, the pictures are grayscale photographs of Ekman faces [43] with angry or fearful expressions. Faces are counterbalanced for sex and emotional valence. In the control condition, the pictures contain geometric shapes (circles, horizontal ellipses, and vertical ellipses). Four blocks per condition, each consisting of one instruction (2 s) and 6 trials (5 s each), are alternately presented. Details are given in Supplementary Figure S5 [see Additional file 1]. The implicit emotion processing task was used identically in the DynaM-OBS study [33].

##### Resting state

A 7-min resting-state scan is acquired during which participants are instructed to keep their eyes open and focus on a fixation cross in the middle of the screen. An identical resting-state scan was collected in the DynaM-OBS study [33]. In the MARP study [56, 57], a 6-min resting-state scan was included.

#### Online questionnaires

The assessment schedule of online questionnaires is outlined in Table 3.

Items of the extended questionnaire battery assess socio-demographic information at month 1 (study baseline), and general health, stressor exposure, mental health, as well as potential psycho-social resilience and risk factors (collectively termed ‘RFs’) at months 1, 6 and 8. RFs included in the battery are assessed as relatively stable styles or traits (i.e., the typical way or tendency in which a person reacts to life experiences). The measures included in the extended questionnaire battery at study baseline will be employed as potential moderators of intervention effects on the primary outcome variables, SR scores and target engagement (see primary research questions in Introduction and Table 1).

The biweekly monitoring questionnaires administered throughout the course of the study assess further information on stressor exposure, mental health, and central RFs necessary to calculate SR scores and target engagement measures as the main outcome variables. To build biweekly SR scores, these questionnaires contain repeated measures of mental health problems (P), assessed by the GHQ-28 [28], and on stressor exposure (E), assessed primarily via a daily hassles list (MIMIS, [44]). Further E measures assessed during the biweekly monitoring, related for example to the COVID pandemic, will be explored for their additional relevance when calculating SR scores (see Table 3).

Target engagement for ReApp is operationalized as the self-reported use frequency of positive cognitive reappraisal (assessed with the items on acceptance, positive reappraisal, putting into perspective, and distancing in the PASS-process questionnaire) and for Imager as the self-reported reward sensitivity (assessed using anticipatory items of the TEPS questionnaire). While RFs included in the extended questionnaire battery are assessed as relatively stable styles, RFs included in the biweekly monitoring were altered to be assessed as modes (i.e., the extent to which the RF was used or experienced in the past two weeks [42]). Complementary and secondary to the biweekly mode assessments, target engagement will also be determined from the corresponding style measures in the extended questionnaire battery.

Finally, biweekly monitoring questionnaires also include additional assessments of self-reported positive appraisals (crisis-related positive appraisals and content-focused perceived positive appraisal). These are not primary measures of target engagement and rather used in moderating analyses and to address tertiary research questions.

Table 5 provides a detailed overview of all questionnaires used in the DynaM-INT study. Validated versions of the questionnaires and their translations to the site-specific languages are used whenever available. An overview of questionnaire validations for the different study languages, as well as the self-developed questionnaires can be found on OSF [63].

#### Video-recorded interview

Each video-recorded interview comprises 13 questions on current mental health problems and recent or future experiences (40 s per recorded answer). Eight questions are based on the four subscales of the GHQ-28 [28] that represent four symptom clusters of psychological distress (somatic complaints, anxiety/insomnia, social dysfunction, and severe depression), with two interview questions per cluster. Four other questions ask about recent positive and negative memories or future expectations, respectively. One additional neutral question serves to establish a baseline for participants’ facial expressivity and vocal features.

Using pretrained open-source algorithms, a comprehensive set of potential DBMs will be extracted from the audio and video material, which roughly fall into four categories: facial expressivity (e.g., positive and negative emotions and overall expressivity), vocal features (e.g., voice pitch and shimmering), movement (e.g., gaze and head movement), and speech content (e.g., the sentiment of answers and word usage). A detailed description of the interview and the analysis will be provided elsewhere.

#### Ecological momentary and physiological assessments

Each EMA questionnaire includes in-the-moment self-assessments of mood (affect), social context, physical context, past event appraisal, and future event appraisal. The morning questionnaire (~1 min) contains questions regarding the last night’s sleep and the phase of the menstrual cycle. The evening questionnaire (~1 min) contains questions regarding the evaluation of the day, as well as stress anticipation of the upcoming day. Supplementary Figures S1 and S2 provide an overview of all assessed EMA items [see Additional file 1].

The Chill + collects four types of EPA-data: photoplethysmogram (PPG, containing infrared and green PPG), galvanic skin response (GSR, containing a signal capped at 2 microSiemens (μS) and one at 20μS), skin temperature (ST) and accelerometer (ACC, in x, y and z direction) data.

##### Feature extraction

Real-time feature extraction and analysis of EMA and EPA data for the purpose of stress-level determination rely on two separate data streams. The upload of EMA data to the Donders Centre for Cognitive Neuroimaging in Nijmegen is implemented in the RADAR-BASE platform. Feature extraction consists of averaging (per EMA beep) all reversed positive affect and all negative affect scores. Negative affect is based on EMA items: “*I feel irritated, anxious*, *insecure and* *sad”*; and positive affect is based on EMA items: “*I feel happy, satisfied and relaxed”*.

The upload of the EPA data is implemented in the DynaMORE chill + app, which enables a Bluetooth connection between the phone and the Chill + device. The DynaMORE chill + app collects 10 min of data prior to the EMA prompt time and sends it to the server hosted by the Donders Centre for Cognitive Neuroimaging in Nijmegen. The feature extraction algorithm considers quality of incoming data, meaning that it will only calculate features based on good quality. The 10 min of data are analyzed in one-minute windows. The results of those separate windows are combined to obtain one value per feature for each data subset of 10 min. Features directly used in the real-time decision algorithm (described below) are the number of spontaneous skin conductance responses, magnitude of spontaneous skin conductance responses, maximum heart rate, and mean heart rate. The number of Chill + button presses (indicating subjectively reported stress moments) is also counted. Details are given in Supplementary Table S2 [see Additional file 1].

##### Threshold calculation

The features from the calibration week during the baseline characterization phase are used to calculate individual EMA/EPA baseline distribution parameters and thresholds for the JITAI triggering during the later intervention phase (booster weeks). For each of the included EMA and EPA features, individualized means and standard deviations are calculated and stored, which are later used to Z-score real-time data for each feature (i.e., relative to the individual baseline distribution).

All EMA features are Z-transformed and averaged into an average EMA Z-score. All EPA features are Z-transformed and averaged into an average EPA Z-score. We then fit a linear regression between the total magnitude of motion based on accelerometer data, and the averaged Z-transformed EPA value. From this regression, the slope and intercept are also stored to residualize the EPA features with respect to motion during real-time analysis in the intervention phase. Finally, EMA Z- scores and motion-corrected average EPA Z-scores are averaged to create a distribution of combined EMA/EPA Z-scores. The initial triggering threshold for EMIs in the first booster week is set at 60% of this distribution (i.e., this value is exceeded in 40% of EMA/EPA beeps in the calibration week), aiming at three interventions per day, with an expected loss of 30% of beeps per day.

##### Real-time decision algorithm

EMA and EPA data collected during the booster weeks in the intervention phase is compared to individual baseline distribution parameters to decide whether an intervention is triggered at that moment. For each new incoming set of EMA/EPA data (i.e., each beep), relevant features are calculated and standardized using the individual baseline distribution parameters (mean and standard deviation of that feature in the calibration week). Z-transformed EMA features are then averaged, resulting in an EMA Z-score for that beep. Z-transformed EPA features are also averaged and then residualized with respect to motion based on the total magnitude of motion obtained from the accelerometers during the same 10-min EPA recording (and using the regression parameters obtained from the calibration week), resulting in the motion-corrected EPA Z-score. Finally, the EMA Z-score and the motion-corrected EPA Z-score are averaged to result in the combined EMA/EPA Z-score.

If there have been less than four interventions triggered for that particular participant in that day, the combined EMA/EPA Z-score is compared to the EMI triggering threshold, which was initially derived from the calibration week data. If this Z-score exceeds the threshold, or if there was a stress button press on the Chill + in the 10 min preceding the EMA questionnaire, an intervention will start. If for a given beep no (high quality) EPA data is available, the decision will be based on EMA features only.

##### Threshold adjustment algorithm

In addition to this algorithm, which is run after each beep, another algorithm which serves to dynamically adapt the triggering threshold is run each night. This second algorithm keeps track of the number of interventions per day and decreases the combined Z threshold at the end of the day by 0.01 if there have been too few interventions (< 3), or raises this threshold by 0.01 if there have been too many (> 3).

#### Ecological momentary interventions

##### Intervention 1: ReApp

The first intervention is targeting positive cognitive reappraisal. In this intervention, participants are asked to think about negative events they experienced or are about to experience in the close future and positively reinterpret them by generating positive reappraisals (e.g., learning from the event, the event has some unexpected positive aspects, advice that they would give to a friend, advice that they would receive from a friend). For details, see [64]. One intervention takes about 2–3 min.

##### Intervention 2: Imager

The second intervention is targeting reward sensitivity via the use of positive mental imagery. In this intervention, participants are asked to think about a pleasurable event that might happen to them during that day and create a mental image of the situation. For details, see [31, 65]. One intervention takes about 2–3 min.

### Data analysis

To evaluate the above research questions, we will conduct two sets of preparatory analyses (addressing feasibility and efficacy), and three sets of main analyses (addressing primary, secondary and tertiary research questions). See Introduction and Table 1.

#### Preparatory feasibility questions (fQ)

The first preparatory analysis addresses the feasibility of the just-in-time-adaptive EMIs that are triggered at moments of high psychological and/or physiological stress. We will consider the technical implementation (fQ1) as well as participant’s adherence (fQ2) and experience (fQ3). These analyses have a descriptive character and may additionally inform exclusion criteria for the main analysis.

To assess the technical implementation of our real-time decision pipeline (fQ1), we will assess the percentage of completed EMA beeps that yielded successful EPA uploads and feature extractions per booster week, the number of minutes per EPA upload in those weeks, and the percentage of triggered interventions per day in each booster week. Further, we will compare the EMA and EPA features of beeps that did and did not trigger an intervention to investigate whether we indeed captured the most stressful moments of the day. Finally, we will examine whether the threshold adjustment algorithm works as expected, by comparing the percentage of triggered interventions per week to the percentage of interventions that would be triggered based on a fixed threshold (i.e., without threshold adjustment algorithm).

To assess adherence (fQ2), we will determine the percentage of completed EMA questionnaires, the percentage of completed triggered interventions, the number of completed self-triggered interventions, the total intervention adherence (i.e., the total number of completed triggered and self-triggered interventions), and the time spent using the aRMT application. All adherence measures will be calculated for each booster week separately, as well as summed for all booster weeks. The percentage of completed EMA questionnaires will additionally be calculated for the calibration week.

User experience (fQ3) is assessed with a shortened version of the user version of the Mobile Application Rating Scale (uMARS) questionnaire [52], which is applied as part of the second extended online questionnaire battery in month 6 in the beginning of the follow-up phase (see Table 3) In addition to the general questions on app usability, we will specifically focus on user experience Q1 (“*What changes did you observe, for example, in your mood, in your behavior *etc*., while using the app?*”) and Q2 *(“Did the app help you use skills during relatively stressful periods?”*) for the feasibility research question.

#### Preparatory efficacy questions (eQ)

The second preparatory analyses address intervention effects on participants’ individual stressor reactivity (SR) scores (eQ1) and target engagement (eQ2). Estimating training efficacy forms the basis for our main analyses of effect moderation (below) and will be achieved by comparing outcome scores during the training period (the intervention phase) to the pre-training baseline (the baseline characterization phase; see Fig. 1).

We choose to examine the overall intervention phase as the outcome phase because the mHealth literature suggests different time-courses over which training effects on health and wellbeing may emerge. For example, a recent meta-analysis reports that only 8–12 week-long resilience interventions already affect different measures of resilience [66], but effects are not sustained at short-term (< 3 months post intervention), medium-term (3–6 months post intervention), or long-term follow-ups (> 6 months post intervention). For other health and wellbeing outcomes, there is evidence of incubation effects. The same meta-analysis shows delay benefits for anxiety and stress measures, which were not reduced post intervention but at short-term follow-up. A meta-analysis of mHealth interventions also reports increasing estimated effect sizes on health outcomes with prolonged follow-up (up to 9 months) [67]. Considering that our resilience operationalization via SR scores aims to improve on previous resilience measures [12] and involves residualized mental health outcomes, effects in the present study may follow either pattern. The use of novel EMIs with a JITAI element in the present study adds further uncertainty. Intervention effects on SR scores and target engagement may thus emerge already after weeks or only after months of training.

We will estimate intervention effects using linear mixed models with repeated SR or target engagement measures (as either modes or styles) as endpoints, comparing measurements that are part of the baseline to those derived during the intervention training period. Long-term follow-up measurements will be treated separately. Our hypothesis is that participants develop lower SR scores and higher target engagement during the interventions.

#### Primary research questions (pQ)

Our primary analysis goal is to assess whether variables (RFs) assessed at study baseline moderate (predict) the effect of ReApp, Imager, or both interventions on SR scores (pQ1) and target engagement measures (pQ2). We will address the pQ1 and pQ2 hypotheses statistically by evaluating the interaction between a given baseline variable and the respective intervention effect estimate, based on the efficacy questions (eQ). Depending on the strength of moderation, training effects may only be detected for a subgroup of participants (see e.g., [64]), such that group-level efficacy is not a prerequisite for addressing these primary research questions. While many baseline variables qualify as potential moderators, the most important ones are the self-reported use frequency of positive cognitive reappraisal for the ReApp intervention, and self-reported reward sensitivity for the Imager intervention (see Online Questionnaires for definition of variables). We hypothesize that lower baseline levels of these resilience factors will be associated with stronger effects of the respective intervention on SR scores (pQ1) and on target engagement (pQ2).

Regarding the potential moderating influence of other psychosocial and neurobiological RFs, the exact analysis plan will depend on the results of the corresponding analyses in our DynaM-OBS observational study [33], which we use as a discovery sample to derive hypothesized moderators and strength of hypotheses (e.g., secondary, tertiary, exploratory).

Given that the two EMIs have differing mechanistic targets, we will first evaluate moderation effects separately in each of the intervention groups. It is also possible that both interventions have unifying moderators, such as PAS. Following separate analyses, if we observe or hypothesize a joint mechanism (such as ultimate effect mediation in both interventions by increases in PAS, see Introduction), we will therefore pool participants over both interventions for combined efficacy and moderation analyses, maximizing analysis power. On the contrary, if we observe or hypothesize potentially differential results, we may instead contrast the two interventions for their main effects and effect moderation. As effect sizes in intervention comparisons are typically relatively small and result in power issues, we consider the latter analyses exploratory.

The above-described linear mixed models represent omnibus analyses of outcome measures across the entire intervention training period. They may thus be followed by post-hoc contrasts of individual measurement time points within the mixed-model framework, allowing us to explore sensitive periods for intervention effects.

##### Supplemental analysis approaches

Next to the above outlined moderation analyses using interaction terms, we will also examine simpler prospective associations between baseline variables of interest and repeated SR score measurements in separate regression models. We aim to replicate associations found in DynaM-OBS [33], and also to compare intervention-related associations in DynaM-INT with associations in natural time-courses in DynaM-OBS. Further analyses may involve DynaM-OBS data [33] as an informal control condition against which the effects of the interventions in DynaM-INT can be assessed. Finally, we will also employ the DynaM-OBS study [33] to explore the applicability of more complex time-series analyses, and to examine the relationship between the different positive appraisal-related measures beyond positive cognitive reappraisal frequency in DynaM-OBS and then try to replicate the result in DynaM-INT.

#### Secondary research question (sQ1)

Our secondary research question is whether the anticipated reductions in stressor reactivity are preceded or accompanied by the anticipated increases in target engagement (sQ1), which would suggest that the interventions work via the targeted resilience mechanisms. To address this question, we will employ linear mixed models for SR-target engagement covariance and lagged associations, respectively. Again, DynaM-OBS [33] results will be consulted to inform the modelling of more complex time-series analyses for example between positive cognitive reappraisal and SR, such as the size of the time lag associations.

#### Tertiary research questions (tQ1-tQ5)

The assessments in DynaM-INT employ various tests potentially suitable to measure PAS. These include the following self-report instruments: Perceived Positive Appraisal Style Scale – process-focused (PASS-process) [45], Perceived Positive Appraisal Style Scale – content-focused (PASS-content) [45], self-generated questions on Crisis-related positive appraisals [63], an optimism questionnaire [48], a control questionnaire [47], and a self-efficacy questionnaire [46] (Table 5). For our tertiary research questions, we will examine their relation to stressor reactivity, target engagement, and potential changes over the study period (tQ1-5) using measurements from the relevant time points.

These questionnaires are employed in the extended questionnaire batteries administered at the baseline characterization and follow-up phases. The PASS-process and PASS-content are additionally included in the biweekly online monitoring questionnaires. A non-questionnaire test is the situation-focused volitional reappraisal fMRI task, as administered in the baseline characterization phase, which has also been employed in earlier studies, including DynaM-OBS, serving to establish the PAS construct and to test its relationship to resilience [33, 57, 68]. These earlier data sets are being used to specify the optimal PAS measure to be used in DynaM-INT before conducting PAS-related analyses in this data set.

#### Additional analyses

##### Digital biomarkers from audiovisual recordings

To obtain more objective and sensitive indicators of participants’ mental health problems, we aim to identify digital biomarkers of mental health (DBMs) from the audiovisual data derived from participants’ video-recorded interviews. The interviews are completed at four timepoints throughout the study. Using pre-trained open-source algorithms, features that represent potential DBMs, such as voice pitch, will be extracted from the recordings. Subsequently, we will use machine learning-based analyses such as feature selection to identify those features that best align with self-reported GHQ scores in a data-driven fashion. Next to convergent validity with the GHQ, we will also consider discriminant validity to other questionnaires, test–retest reliability, and consistency across multiple analysis approaches.

In a second step, we aim to combine the identified features to DBM-based P scores and use them to calculate DBM-based SR scores, which can complement the primary, fully questionnaire-based SR as an additional outcome in addressing the above hypotheses. For example, we will investigate intervention effects on DBM-based SR scores, whether the same RFs that predict questionnaire-based SR also predict DBM-based SR, and whether those RFs that are not measured via self-report questionnaires, such as fMRI task-based activation or biological data from the bio-samples, show stronger associations with the DBM-based than questionnaire-based SR scores. Next to using identified DBMs in a complementary outcome measure, we will also explore how potential DBMs relate to the main questionnaire-based SR as predictors and whether any features relate to or predict intervention success.

## Discussion

With the DynaM-INT study, we are advancing the field of resilience research by investigating two different just-in-time adaptive interventions (JITAIs) that are targeted at increasing putative resilience factors. The design allows us to investigate the feasibility of just-in-time EMIs, triggered at moments of high psychological and physiological stress in real life. The multimodal baseline characterization further enables us to identify predictors for the effects of each of the interventions on stressor reactivity and target engagement. At the same time, the dense longitudinal measures allow us to investigate whether the JITAIs are followed by reductions in stressor reactivity and increases in target engagement over time. The DynaM-INT study thereby aims to inform future research about which parameters are important to consider in future studies testing the efficacy of these interventions. Moreover, the DynaM-INT study yields a rich database that can be shared with other researchers in the field of resilience research.

### Supplementary Information

**Additional file 1:** **Table S1.** Beep schedule. **Figure S1.** Design of the different assessment weeks. **Figure S2.** EMA content. **Table S2.** Real-time features. **Figure S3.** Design of the reward sensitivity task. **Figure S4.** Design of the Situation-focused volitional reappraisal task. **Figure S5.** Design of the implicit emotion processing task (Faces task). **Table S3.** Remuneration schedule.

## Data Availability

Self-generated questionnaires are available at OSF [44].

## Abbreviations

3T: 3 Tesla
AP: Anterior-posterior
BOLD: Blood oxygen level dependent
d: Day
DBM: Digital biomarkers of mental health derived from audiovisual recordings
DNA: Deoxyribonucleic acid
DynaM-INT: Dynamic Modelling of Resilience-Interventional Study
DynaM-OBS: Dynamic Modelling of Resilience-Observational Study
E: Experienced stressors
EMA: Ecological momentary assessment
EMI: Ecological momentary intervention
EPA: Ecological physiological assessment
EPI: Echoplanar imaging
FLAIR: Fluid-attenuated inversion recovery
(f)MRI: (Functional) magnetic resonance imaging
FOV: Field of view
FRESHMO: Frequent stressor and mental health monitoring
GHQ: General health questionnaire
HAWIE: Hamburg Wechsler intelligence test for adults
h: Hour
HR: Heart rate
IAPS: International affective picture system
ID: Identifier
JITAI: Just-in-time adaptive intervention
MB: Multiband
MID: Monetary incentive delay task
M.I.N.I.: Mini-International Neuropsychiatric Interview
ml: Milliliter
M: Month
ms: Millisecond
NIS: Israeli Shekel
nr: Number
P: Mental health problems
PA: Posterior-anterior
PAS: Positive appraisal style
PASS-content: Perceived positive appraisal style scale, content-focused
PASS-process: Perceived positive appraisal style scale, process-focused
PASTOR: Positive appraisal style theory of resilience
PLN: Polish Złoty
R > NR: Contrast regulate>no regulate
RF: Resilience or risk factor
s: Second
SR: Stressor reactivity
T1-MPRAGE: Magnetization prepared rapid acquisition with gradient echoes
TE: Echo time
TR: Repetition time
w: Week
