# Supplementary material for: Investigating two mobile just-in-time adaptive interventions to foster psychological resilience: research protocol of the DynaM-INT study
Source: BMC Psychol. 2023 Aug 25;11:245. doi: 10.1186/s40359-023-01249-5 (PMC10464364; doi:10.1186/s40359-023-01249-5)
Supplement: Supplementary file 1 — Additional file 1: Table S1. Beep schedule. Figure S1. Design of the different assessment weeks. Figure S2. EMA content. Table S2. Real-time features. Figure S3. Design of the reward sensitivity task. Figure S4. Design of the Situation-focused volitional reappraisal task. Figure S5. Design of the implicit emotion processing task (Faces task). Table S3. Remuneration schedule. [file 40359_2023_1249_MOESM1_ESM.docx]

**Investigating two Mobile Just-in-Time Adaptive Interventions to Foster Psychological Resilience: Research Protocol of the DynaM-INT study – Supplementary Information**

Bögemann, S.A.^1,a^, Riepenhausen, A.^2,3,a^, Puhlmann, L.M.C.^4,5,a^, Bar, S.^6^, Hermsen, E.J.C.^1^, Mituniewicz, J.^7^, Reppmann, Z.C.^2^, Uściƚko, A.^7^, van Leeuwen, J.M.C.^1^, Wackerhagen, C.^2^, Yuen, K.S.L.^4,8^, Zerban, M.^8^, Weermeijer, J.^9^, Marciniak, M.A.^10,11^, Mor, N.^6,12^, van Kraaij, A.^13^, Köber, G.^14,15^, Pooseh, S.^15^, Koval, P.^16^, Arias-Vásquez, A.^1^, Binder, H.^14,15^, De Raedt, W.^17^, Kleim, B.^10,11^, Myin-Germeys, I.^9^, Roelofs, K.^18,19^, Timmer, J.^15,20,21^, Tüscher, O.^4,22^, Hendler, T.^6,12,23,24,b^, Kobylińska, D.^7,b^, Veer, I.M.^25,b^, Kalisch, R.^4,8,b^, Hermans, E.J.^1,b^, Walter, H.^2,3,b^

^1^ Donders Institute for Brain, Cognition and Behaviour, Radboud university medical center, Nijmegen, The Netherlands

^2^ Research Division of Mind and Brain, Department of Psychiatry and Neurosciences CCM, Charité - Universitätsmedizin Berlin, Corporate Member of Freie Universität Berlin, Humboldt-Universität zu Berlin, and Berlin Institute of Health, Berlin, Germany

^3^ Faculty of Philosophy, Berlin School of Mind and Brain, Humboldt-Universität zu Berlin, Berlin, Germany

^4^ Leibniz Institute for Resilience Research (LIR), Mainz, Germany

^5^ Max Planck Institute for Human Cognitive and Brain Sciences, Leipzig, Germany

^6^ Sagol Brain Institute, Tel Aviv Sourasky Medical Center, Tel Aviv, Israel.

^7^ Faculty of Psychology, University of Warsaw, Warsaw, Poland

^8^ Neuroimaging Center (NIC), Focus Program Translational Neuroscience (FTN), Johannes Gutenberg University Medical Center, Mainz, Germany

^9^ Center for Contextual Psychiatry, Department of Neurosciences, KU Leuven, Leuven, Belgium

^10^ Division of Experimental Psychopathology and Psychotherapy, Department of Psychology, University of Zurich, Zurich, Switzerland

^11^ Department of Psychiatry, Psychotherapy and Psychosomatics, Psychiatric University Hospital (PUK), University of Zurich, Zurich, Switzerland

^12^ Sackler Faculty of Medicine, Tel Aviv University, Tel Aviv, Israel

^13^ OnePlanet Research Center, Wageningen, The Netherlands

^14^ Institute of Medical Biometry and Statistics, Faculty of Medicine and Medical Center – University of Freiburg, Freiburg, Germany

^15^ Freiburg Center for Data Analysis and Modelling, University of Freiburg, Freiburg, Germany

^16^ Melbourne School of Psychological Sciences, The University of Melbourne, Vic 3010, Australia.

^17^ Life Sciences Department, imec, Leuven, Belgium

^18^ Center for Cognitive Neuroimaging, Donders Institute for Brain Cognition and Behaviour, Radboud University, Nijmegen, The Netherlands

^19^ Behavioural Science Institute, Radboud University, Nijmegen, The Netherlands

^20^ Institute of Physics, University of Freiburg, Freiburg, Germany

^21^ Signalling Research Centres BIOSS and CIBSS, University of Freiburg, Freiburg, Germany

^22^ Department of Psychiatry and Psychotherapy, Johannes Gutenberg University Medical Center, Mainz, Germany

^23^ School of Psychological Science, Tel Aviv University, Tel Aviv, Israel

^24^ Sagol School of Neuroscience, Tel Aviv University, Tel Aviv, Israel

^25^ Department of Developmental Psychology, University of Amsterdam, Amsterdam, The Netherlands

^a,b^ equal contribution

Corresponding Author:

Sophie Bögemann

ORCID: 0000-0001-9382-0769

Donders Institute for Brain, Cognition, and Behaviour

Radboud university medical center

Kapittelweg 29, 6525 EN Nijmegen, The Netherlands

sophie.bogemann@donders.ru.nl

1. **Daily-life Assessments**
   1. Beep Schedule

| **Day 1** | **Day 2** | **Day 3** | **Day 4** | **Day 5** | **Day 6** |
| --- | --- | --- | --- | --- | --- |
| 7:54 | 8:02 | 7:41 | 7:57 | 8:00 | 7:31 |
| 10:03 | 9:02 | 9:32 | 9:05 | 9:17 | 9:33 |
| 10:41 | 11:13 | 11:12 | 11:24 | 11:27 | 10:32 |
| 12:46 | 13:26 | 13:04 | 12:28 | 12:59 | 12:33 |
| 13:56 | 13:53 | 13:47 | 13:52 | 13:32 | 14:34 |
| 15:13 | 16:21 | 16:28 | 16:07 | 15:08 | 15:12 |
| 16:32 | 17:21 | 17:06 | 16:33 | 17:16 | 17:37 |
| 18:56 | 18:13 | 18:54 | 19:24 | 18:15 | 18:56 |
| 20:14 | 20:17 | 20:49 | 20:11 | 20:00 | 20:25 |
| 22:21 | 21:05 | 22:29 | 21:59 | 22:09 | 22:20 |

**Table S1. Beep schedule.** The beep schedule is fixed for the calibration week. During the intervention phase, the beep schedule is repeated in the background continuously. The starting day of each booster week is flexible, meaning that beep schedule during the booster weeks may start on any of day 1-6.

- 1. Assessments Per Week


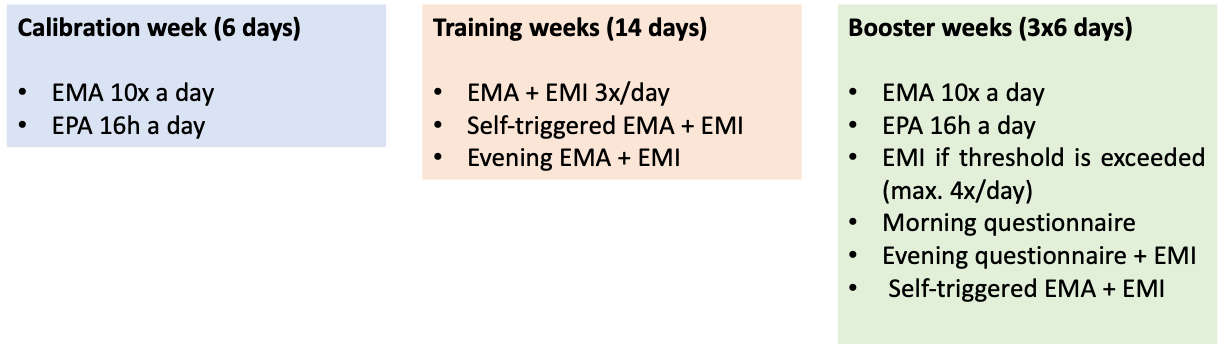


**Figure S1. Design of the different assessment weeks.** During the intervention phase, participants are encouraged to keep practicing the intervention via self-triggered EMA + EMI modules in the SEMA3 app. The encouraged practice weeks have similar assessments as the regular training weeks. Abbreviations: ecological momentary assessments (EMA), ecological physiological assessments (EPA), ecological momentary interventions (EMI).

- 1. EMA questionnaire


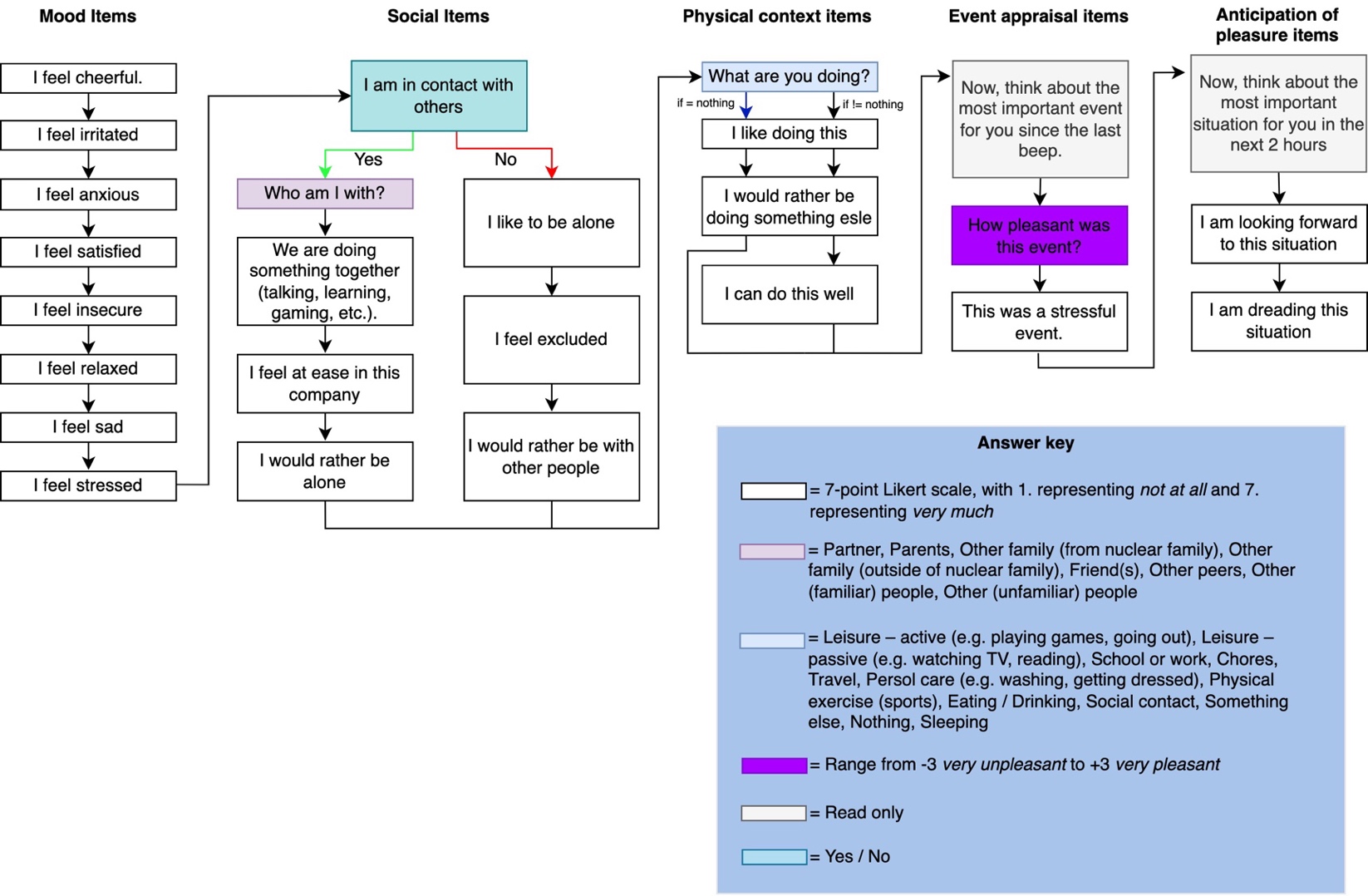


**Figure S2. EMA content.** Additional morning and evening questionnaires are answered after waking up, and before going to bed, respectively. The morning questionnaire included the items: “At what time did you fall asleep?”, “At what time did you wake up?”, “I slept well”, “I feel rested”, “How long did it take you to fall asleep?”, and “Which phase of the menstruation cycle are you currently in?”. The evening questionnaire included the items: “This was a bad day for me”, “This was a nice day”, “Today I spent about … minutes exercising”, “I expect stressful events tomorrow”, “I am confident I can cope with tomorrow's stressful events”, “I expect pleasant events tomorrow”, and “I am looking forward to the pleasant things I am expecting tomorrow”.

- 1. Real-time Features

| Feature | Description | Used in real-time decision tree? |
| --- | --- | --- |
| EMA |  |  |
| PA_rev | Reversed positive affect | Yes |
| NA | Negative affect | Yes |
|  |  |  |
| EPA |  |  |
| SCnumber | Number of spontaneous skin conductance responses | Yes |
| SCmagnitude | Magnitude of spontaneous skin conductance responses | Yes |
| SClevel | Level of skin conductance | No |
| SCnumberofsamples | Received number of samples | No |
| SChighqualitypercentage | Amount of high-quality data received. Value between 0 and 1. | No |
| PPG-HRmaximum | Maximum heart rate | Yes |
| PPG-HRmean | Mean heart rate | Yes |
| PPG-HRminimum | Minimum heart rate | No |
| PPGnumberofsamples | Received number of samples |  |
| STslope | The slope of skin temperature | No |
| STmedian | Median skin temperate | No |
| STstd | Skin temperate standard deviation | No |
| STnumerofsamples | Received number of samples | No |
| SThighqualitypercentage | Amount of high-quality data received. Value between 0 and 1. | No |
| ACCmagnitude | Magnitude of movement (in all directions) | Indirectly |
| ACCnumberofsamples | Received number of samples | No |
| PIEnumberofsamples | Received number of samples | No |
| PIEeventcount | Amount of button presses | Yes |

**Table S2. Real-time features.** EMA and EPA features calculated in real-time during the calibration and booster weeks.

1. **Design of Neuroimaging Tasks**
   1. Reward Sensitivity


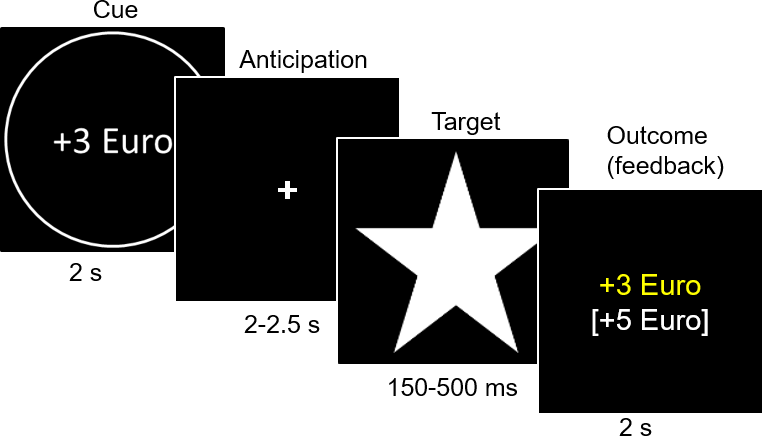


**Figure S3. Design of the reward sensitivity task.** Each trial started with a 2-seconds cue indicating the incentive condition (+3 €, +0.5 €, ±0 €, -0.5 €, -3 €), followed by an anticipation phase of 2 to 2.5s. Subjects had to press the right button when the target (a white star) appeared on the screen. Target duration was adapted based on past task performance in a range from 150 and to 500 ms. Each trial ended with a 2-seconds numeric feedback on subjects’ trial outcome (gained amount in yellow or lost amount in red) and overall outcome (accumulated total amount). As an example, a correct +3 € trial is shown. Figure by Kampa et al. [1], adopted with permission from the authors.

- 1. Situation-focused Volitional Reappraisal


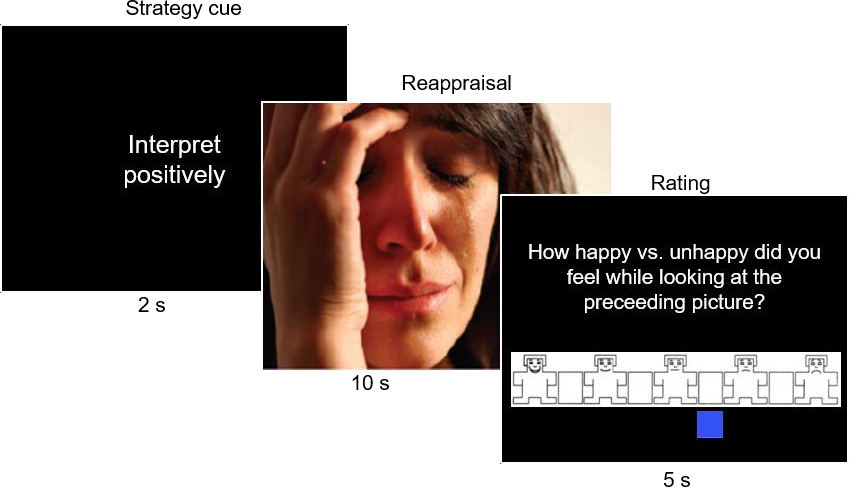


**Figure S4. Design of the Situation-focused volitional reappraisal task.** Subjects had to either make their emotional state more positive by positively reinterpreting a depicted situation (Reappraisal or R trials) or not (No Reappraisal or NR trials). Each trial started with a 2-seconds strategy cue word (“interpret positively” in R or “view image” in NR trials), followed by a 10-seconds presentation of a positive (Pos), neutral (Neu) or negative (Neg) picture, during which the strategy was to be applied. Each trial ended with an emotional state rating from 0 to 5 for 5 s. As an example, a Neg/R trial is shown. The depicted image is retrieved from the EmoPicS (Wessa et al., 2010) and was already published in Kanske et al. (2011). It is used as a placeholder for emotional images here but is not part of the stimulus set of Task 5. Figure by Kampa et al. [1], adopted with permission from the authors.

- 1. Implicit Emotion Processing (Faces Task)


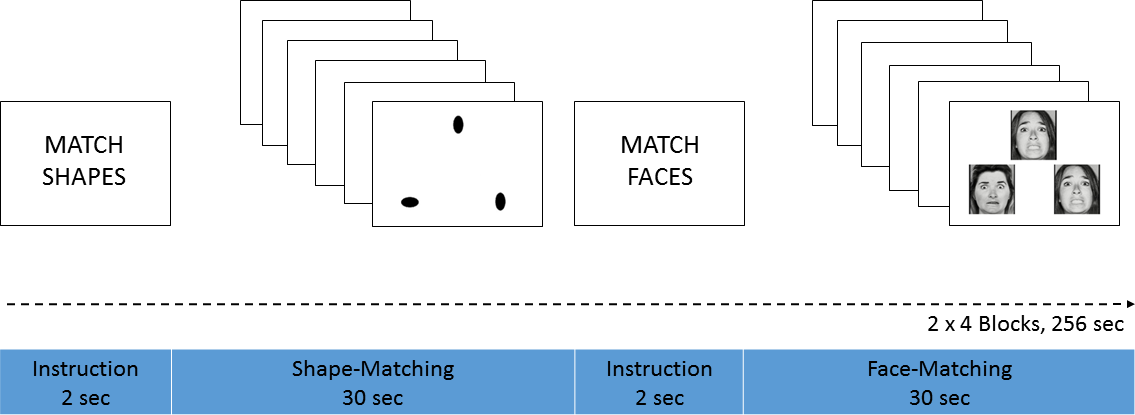


**Figure S5. Design of the implicit emotion processing task (Faces task).** Each block started with a brief instruction. In all conditions, participants were instructed to identify which one of the two stimuli in the bottom is identical to the stimulus in the top. During the shape-matching blocks, stimuli contained of vertical and horizontal ellipses, while during the face-matching blocks, stimuli consisted of photographs of male and female faces expressing anger or fear. Figure by Sacu et al. [2], adopted with permission from the authors.

1. **Remuneration**

|  |  | **Baseline phase** | | | | | | | **Ecological momentary intervention phase** | | | | | | | | | | | | | | | | **Follow-up phase** | | | | | | | | | | | | |
| --- | --- | --- | --- | --- | --- | --- | --- | --- | --- | --- | --- | --- | --- | --- | --- | --- | --- | --- | --- | --- | --- | --- | --- | --- | --- | --- | --- | --- | --- | --- | --- | --- | --- | --- | --- | --- | --- |
|  |  | Month 1 | | | | |  | | Month 2 | | | | Month 3 | | | | Month 4 | | | | Month 5 | | | | Month 6 | | | | Month 7 | | | | Month 8 | | | | |
|  |  | Week | | | | |  | | Week | | | | Week | | | | Week | | | | Week | | | | Week | | | | Week | | | | Week | | | | |
|  |  | 1 | 2 | 3 | 4 | 1 | | 2 | | 3 | 4 | 1 | | 2 | 3 | 4 | 1 | 2 | 3 | 4 | 1 | 2 | 3 | 4 | 1 | 2 | 3 | 4 | 1 | 2 | 3 | 4 | | 1 | 2 | 3 | 4 |
|  | Berlin (EUR) | 52^#^ + MID win |  |  |  |  | |  | |  |  |  | |  |  |  |  |  |  |  |  |  |  | 189,10^#^ |  |  |  | 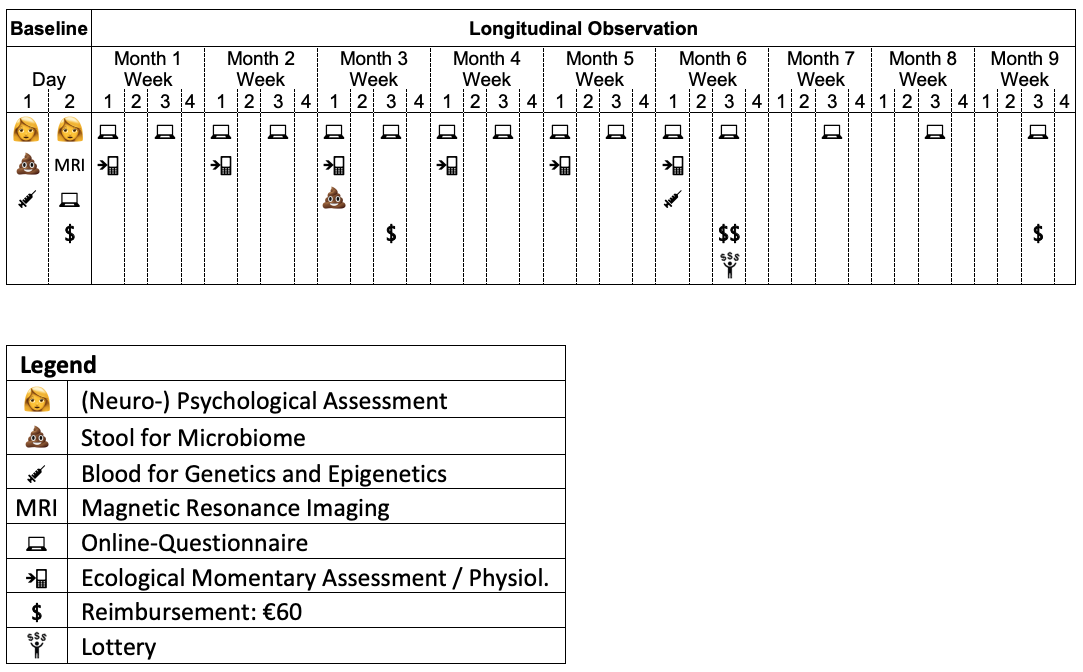* |  |  |  |  | |  |  |  | 48,90^#^ + 50* |
|  | Mainz (EUR) | 100^#^ + MID win |  |  |  |  | |  | |  |  |  | |  |  |  |  |  |  |  |  |  |  | 120^#^ |  |  |  | 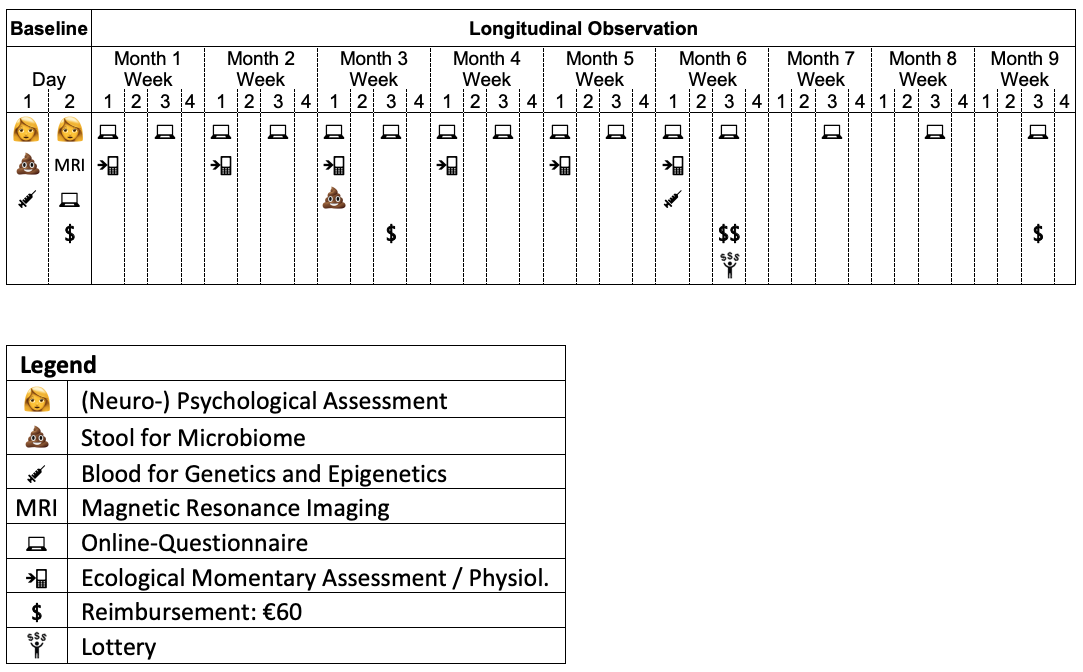* |  |  |  |  | |  |  |  | 120^#^ |
|  | Nijmegen (EUR) | 100 + MID win |  |  |  |  | |  | |  |  |  | |  |  |  |  |  |  |  |  |  |  | 120 |  |  |  | 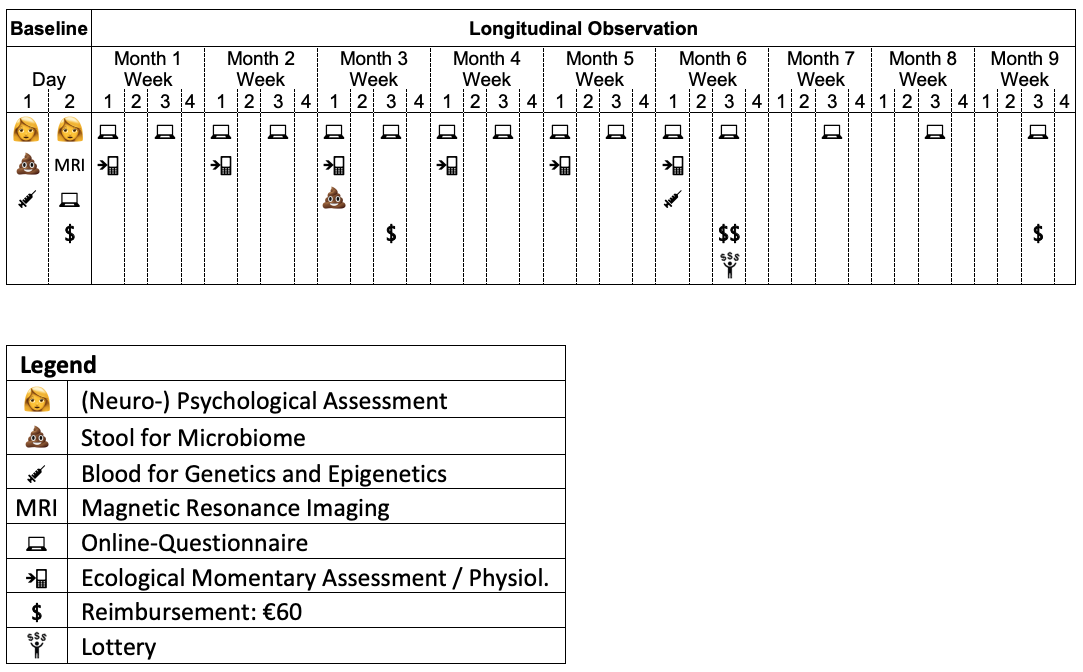* |  |  |  |  | |  |  |  | 120 |
|  | Tel Aviv (NIS) | 275 + MID win |  |  |  |  | |  | |  |  |  | |  |  |  |  |  |  |  |  |  |  | 575 |  |  |  | 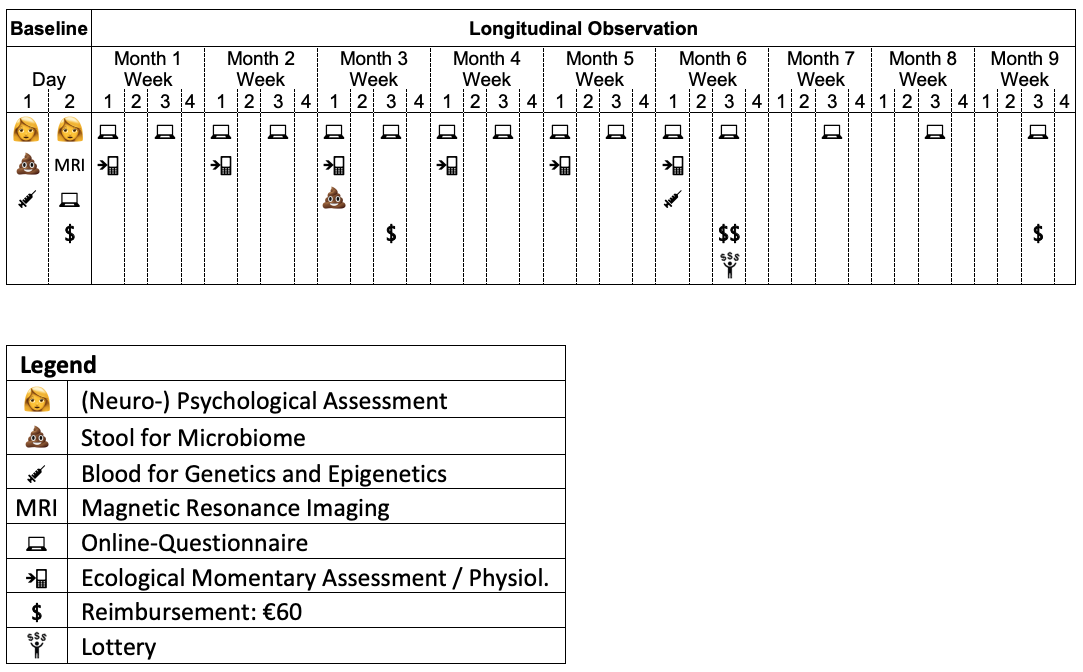* |  |  |  |  | |  |  |  | 185 + 175* |
|  | Warsaw (PLN) |  |  |  |  |  | |  | |  |  |  | |  |  | 700^#^ + MID win |  |  |  |  |  |  |  |  |  |  |  | 390^#^ +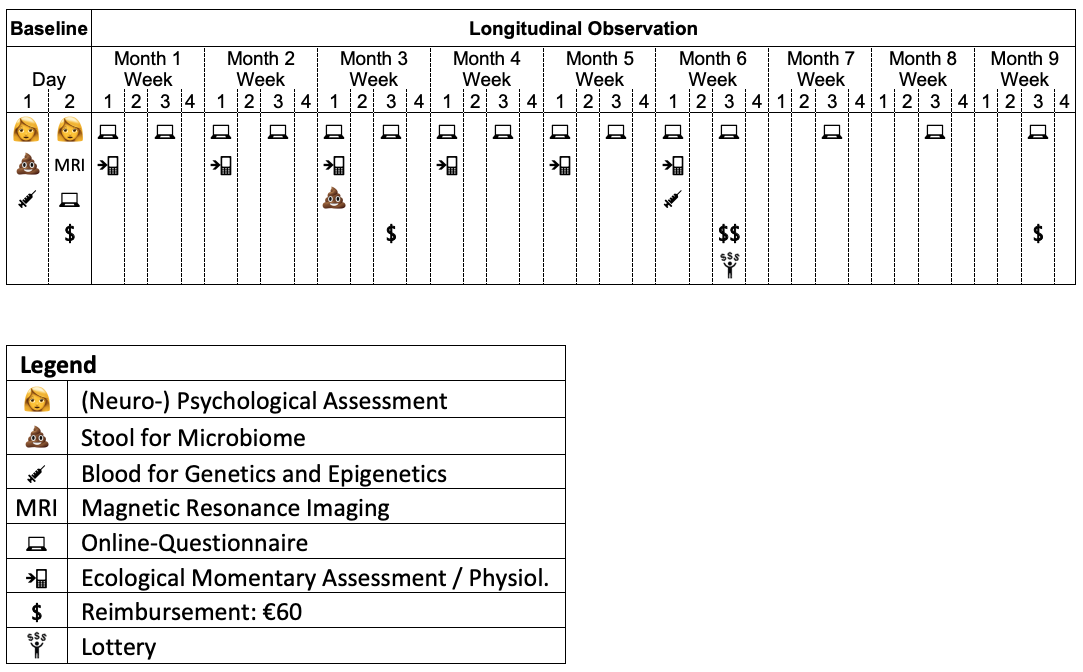* |  |  |  |  | |  |  |  | 110^#^ |

| **Legend** | |
| --- | --- |
| 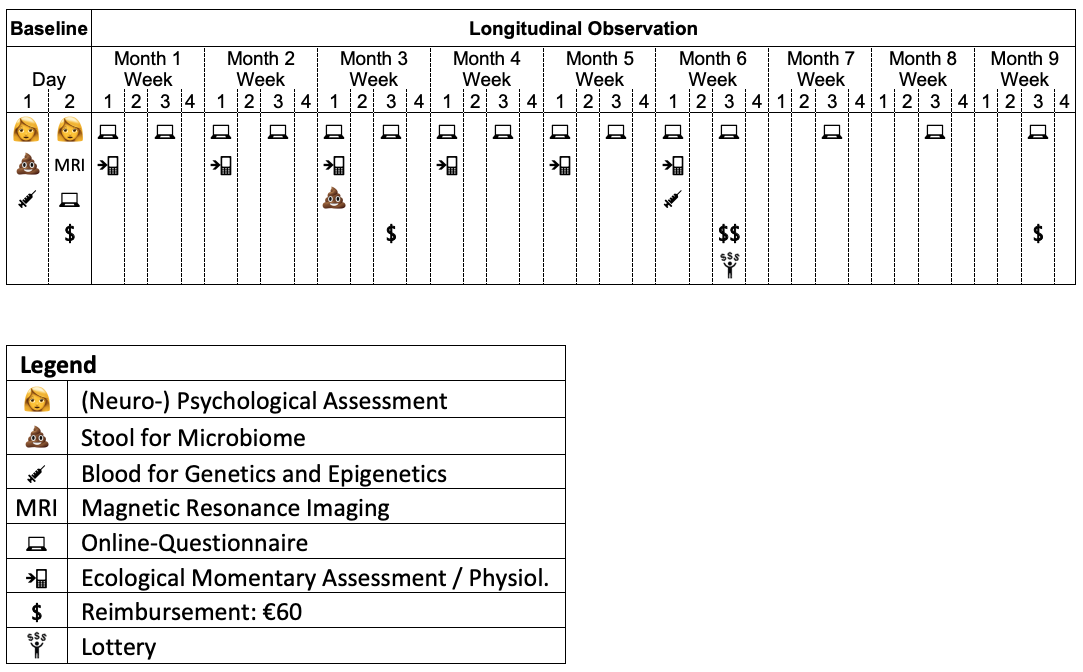 | Lottery (up to 5 vouchers of 100 EUR / 400 NIS / 400 PLN per site) |

**Table S3. Remuneration schedule.** Participants receive 340 EUR / 1200 NIS / 1200 PLN for complete study participation. Further, they receive about 10 EUR / 40 NIS / 40 PLN on average during the reward task (monetary incentive delay task, MID), and are included in a lottery to win one out of 5 times 100 EUR / 400 NIS in Berlin, Main, Nijmegen and Tel Aviv, or one time 400 PLN in Warsaw if they accomplish all assessments until (and including) month five, week three. In addition, Berlin and Tel Aviv include a monetary bonus if all assessments are accomplished (indicated with a *). In Berlin, Mainz and Warsaw, the height of the actual payment was dependent on the amount of completed study assessments (indicated with a ^#^). The amounts in the table indicate the maximum amount.

1. **References**

1. Kampa M, Schick A, Yuen K, Sebastian A, Chmitorz A, Saase V, et al. A Combined Behavioral and Neuroimaging Battery to Test Positive Appraisal Style Theory of Resilience in Longitudinal Studies. bioRxiv. 2018;:470435.

2. Sacu S, Wackerhagen C, Erk S, Romanczuk-Seiferth N, Schwarz K, Schweiger JI, et al. Effective connectivity during face processing in major depression – distinguishing markers of pathology, risk, and resilience. Psychol Med. 2022;:1–13.
